# ATRA upregulates OTUD6B to recruit CD8^+^ T cells to suppress colorectal liver metastasis by stabilizing DDX5/STAT3/CXCL11 axis

**DOI:** 10.1038/s41419-025-07837-0

**Published:** 2025-07-12

**Authors:** Jinglei Li, Kunpeng Huang, Bing Yang, Xia Hu, Bosheng Mei, Xiang Cheng, Xin Zhong, Chuyi Cao, Zihan Chen, Hui Wang, Jinxiang Zhang

**Affiliations:** 1 Department of Emergency Surgery, Union Hospital, Tongji Medical College, Huazhong University of Science and Technology, Wuhan, China; 2 Department of Gastroenterology, Second Affiliated Hospital of Nanchang University, Nanchang, China; 3 Cancer Center, Union Hospital, Tongji Medical College, Huazhong University of Science and Technology, Wuhan, China; 4 Department of Medical Genetics, Basic School of Tongji Medical College, Huazhong University of Science and Technology, Wuhan, China

**Keywords:** Metastasis, Cancer microenvironment

## Abstract

OTU deubiquitinase 6B (OTUD6B) study in tumors is gradually increasing; however, studies on the role of OTUD6B in colorectal cancer (CRC) are rare. OTUD6B was overexpressed in some human CRC and liver metastasis samples. Although OTUD6B facilitated migration and invasion in CRC cells, it exhibited opposite effects on liver metastasis in immunodeficient and immunocompetent mice. We demonstrated that Otud6b enhanced metastasis in nude mice, but it recruited more CD8^+^ T cell infiltration in colorectal liver metastasis (CRLM) mouse model of C57BL/6J to inhibit CRLM through upregulating Cxcl11. Furthermore, we demonstrated that OTUD6B deubiquitinated and stabilized DDX5. Ectopically expressed DDX5 facilitated transcription factor STAT3 activation by resolving the RNA G-quadruplex structure of STAT3, resulting in a higher level of CXCL11 transcription and an increase in tumor-infiltrating CD8^+^ T cells. All-trans retinoic acid inhibited CRLM by upregulating OTUD6B.

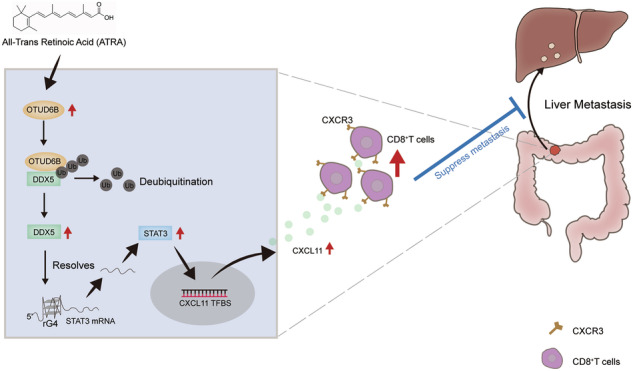

## Introduction

Colorectal cancer (CRC) is the third most common cancer and the second leading cause of cancer death globally [1]. Metastasis is the leading cause of death among patients with CRC, and the liver is the most common organ for CRC metastasis [2, 3]. The liver metastatic cascade of CRC is a complicated, multifactorial, and multistep biological process; thus, investigating the molecular mechanisms regulating metastasis is crucial for treating patients with CRC [4].

OTU deubiquitinase 6B (OTUD6B) belongs to the ovarian tumor family deubiquitinases [5]. OTUD6B exhibits diverse roles in various tumors. OTUD6B suppressed hepatocellular carcinoma metastasis, involving a feedback loop consisting of OTUD6B, pVHL, and HIF-1α [6]; CircPTPN12 bolstered the assembly of the PDLIM2/OTUD6B complex and inhibited hepatocellular carcinoma progression by PDLIM2/NF-κB pathway [7]. And OTUD6B was identified as a potent deubiquitinase of β-TrCP that suppressed esophageal squamous cell carcinoma progression through the OTUD6B-β-TrCP-SNAIL axis [8]. However, OTUD6B facilitated breast cancer cell survival by supporting KIFC1 expression [9]. And OTUD6B promoted lung adenocarcinoma progression by stabilizing RIPK1 [10]. However, the role of OTUD6B in CRC liver metastasis is unknown.

RNA helicase DDX5 functions as a transcriptional coactivator of many oncogenic transcription factors and has an essential role in RNA metabolism. It is a coactivator of STAT3 and upregulates STAT3 downstream genes [11]. Additionally, the recent study demonstrated that RNA helicase DDX5 resolved a secondary RNA structure called G-quadruplex, located in the 5’ untranslated region (5’UTR) of STAT1 mRNA, enabling its translation. G-quadruplexes are four-stranded structures formed in guanine (G)-rich sequences, and their presence in 5’UTRs of mRNAs affects post-transcriptional regulation of gene expression [12, 13].

CXCL11 binds to two different chemokine receptors, CXCR3 and CXCR7. CXCL9, CXCL10, and CXCL11 are three selective ligands that bind to CXCR3; however, CXCR3 binds to CXCL11 with higher affinity than CXCL9 or CXCL10 [14]. Additionally, CXCL11 can bind to another chemokine receptor, CXCR7, which can be engaged by CXCL12. However, CXCR7 has a 10- to 20-fold reduced affinity for CXCL11 than CXCL12 [15]. CXCL11 has potent antitumor activity in vivo by facilitating the infiltration of CD8^+^ T lymphocytes. A positive correlation was observed between CXCL11 and tumor-infiltrating CD8^+^ T cells in mice subjected to genetically modified CXCL11-EL4 T cell lymphoma cells. Depletion of CD8^+^ T cells in vivo completely abrogated the antitumor efficacy of CXCL11 [16].

This study revealed that OTUD6B facilitated CRC liver metastasis in immunodeficient mice. However, it inhibitd CRC liver metastasis in immunocompetent mice. OTUD6B recruited CD8^+^ T cells to enhance the immune response by stabilizing the DDX5/STAT3/CXCL11 axis in CRC liver metastasis. ATRA upregulated OTUD6B expression to inhibit CRC liver metastasis.

## Results

### OTUD6B is overexpressed in CRC and positively correlated with survival rate

**Fig. 1:**
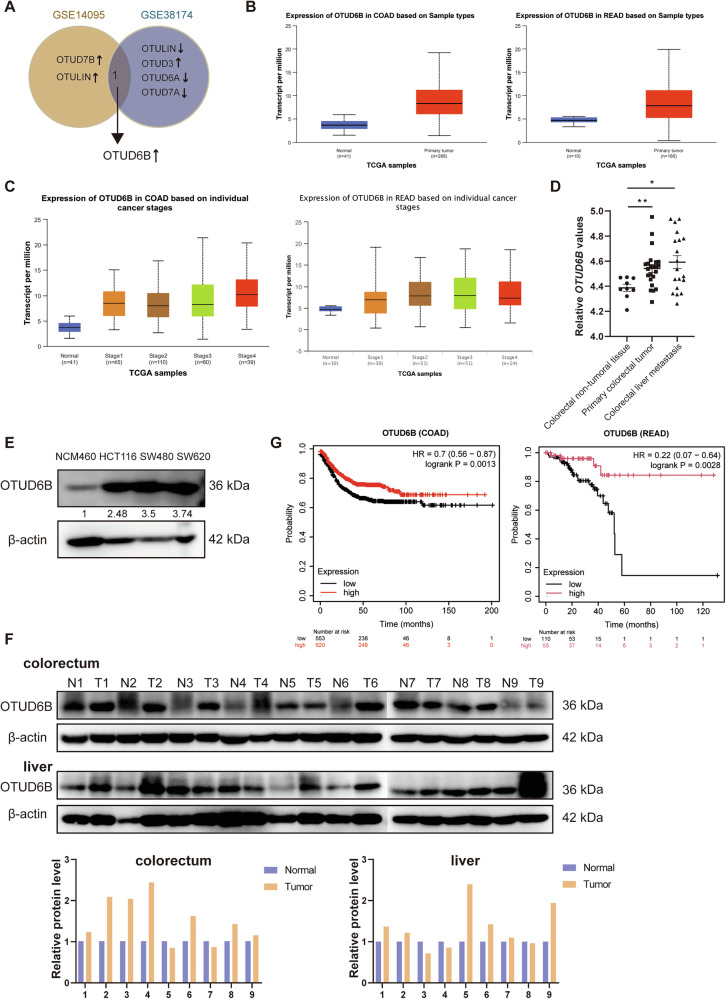
OTUD6B is overexpressed in CRC and positively correlated with survival rate. **A** Only OTUD6B changed in the same direction in GSE14095 and GSE38174. **B** OTUD6B expression was analyzed in COAD and READ according to the TCGA database retrieved from the UALCAN database. **C** OTUD6B expression was analyzed in different stages of COAD and READ using the UALCAN database. **D** OTUD6B expression was analyzed in the primary colorectal tumor and colorectal liver metastasis at GSE81582. **E** Representative immunoblots for OTUD6B and β-actin expression in human CRC cells and normal colonic epithelial cells. **F** Representative immunoblots for OTUD6B and β-actin expression in 9 paired CRC and liver tissues and their adjacent normal tissues. N normal, T tumor. **G** Survival analysis between patients with OTUD6B high- and low-expression COAD and READ using kmplot database. Data are expressed as mean ± SEM. A two-sided Student’s *t* test was used for the statistical analysis. *P < 0.05; **P < 0.01.

Our previous study has demonstrated that the OTU family member, OTUD1, inhibited lung cancer progression [17]. Gene Expression Omnibus (GEO) database (GSE14095 and GSE38174) analysis of differentially expressed genes of the OTU family in CRC liver metastasis and normal tissue revealed that only OTUD6B mRNA level was upregulated in both datasets (Fig. 1A). *OTUD6B* mRNA level was significantly upregulated in colon adenocarcinoma (COAD) and rectum adenocarcinoma (READ) samples compared to normal tissues, as indicated by data from the TCGA database accessed through the UALCAN database (Fig. 1B). *OTUD6B* mRNA level was upregulated at various stages in COAD and READ (Fig. 1C). Additionally, *OTUD6B* mRNA expression was upregulated in primary CRC and CRC liver metastasis on GSE81582 (Fig. 1D). OTUD6B protein level was upregulated in all CRC cells compared to normal colonic epithelial cells (Fig. 1E). OTUD6B protein level in colorectal and liver tumors and their respective adjacent normal tissues of nine patients with CRC liver metastasis were detected using Western blot to confirm whether OTUD6B is highly expressed in colorectal liver metastasis (CRLM). OTUD6B protein expression level was significantly higher in most colorectal and liver tumors than in corresponding normal tissues (Fig. 1F). High OTUD6B expression in CRC samples correlated with longer survival in the kmplot database (Fig. 1G). OTUD6B was overexpressed in CRC and liver metastasis, and OTUD6B was positively correlated with survival rate.

### Opposing effects of OTUD6B on CRLM in immunodeficient and immunocompetent mice

**Fig. 2:**
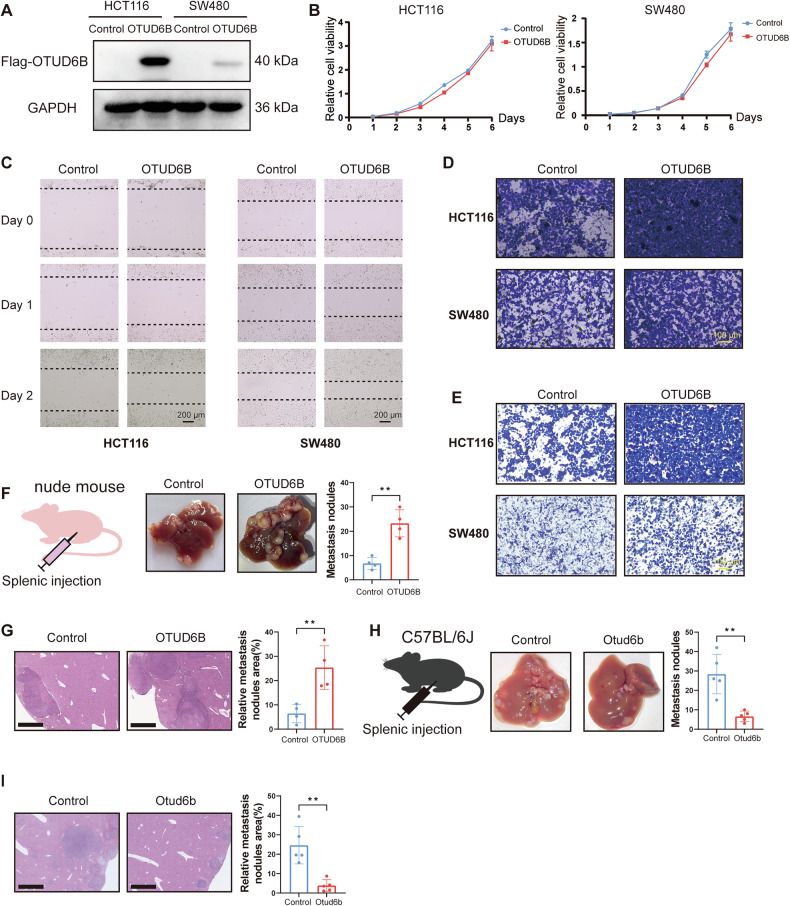
Opposing effects of OTUD6B on CRLM in immunodeficient and immunocompetent mice. **A** OTUD6B-Flag expression level in stable OTUD6B overexpression cell lines (HCT116 and SW480) and the corresponding control using Western blot analysis. **B** Effects of OTUD6B overexpression on the viability of HCT116 and SW480 CRC cells. Cell viability was monitored for 6 days using CCK-8 assay. **C** Effects of OTUD6B overexpression on the migration of HCT116 and SW480 CRC cells using a wound-healing assay. Wound closure was determined at 1- and 2-day time points. Scale bar, 200 µm. **D** Effects of OTUD6B overexpression on cell migration. Control or OTUD6B-overexpressing cells were assayed in Transwell chambers. Migrative potential was assessed after a 24-h incubation. Scale bar, 100 µm. **E** Effects of OTUD6B overexpression on cell invasion. Control or OTUD6B-overexpressing cells were assayed in Transwell chambers with matrigel. Invasive potential was assessed after a 24-h incubation. Scale bar, 100 µm. **F** The schematic diagram showed HCT116 cells to induce CRLM model in nude mice (left). Representative images of liver metastatic tissue and the number of liver metastatic nodules from HCT116 cells with or without OTUD6B overexpression in nude mice (*n* = 4). **G** Representative pictures and quantitative results of H&E staining of liver tissue sections from (**F**). Scale bar, 1 mm. **H** The schematic diagram showed MC38 cells to induce CRLM model in C57BL/6J mice (left). Representative pictures of liver metastatic tissue and the number of liver metastatic nodules from C57BL/6J mice injected with MC38-Control or MC38-Otud6b-overexpression cells (*n* = 5). **I** Representative pictures and quantitative results of H&E staining from (**H**). Scale bar, 1 mm. Data are expressed as mean ± SEM. A two-sided Student’s *t* test was used for the statistical analysis. **P < 0.01.

We subsequently examined the function of OTUD6B in CRC. Stable cell lines with overexpressed OTUD6B were successfully established (Fig. 2A). OTUD6B overexpression exhibited no significant effect on CRC cell proliferation but enhanced cell migration and invasion (Fig. 2B–E). Furthermore, we developed the stable cell lines that knocked down OTUD6B (Supplementary Fig. S1A). OTUD6B knockdown exhibited no significant effect on cell proliferation, consistent with previous observations (Supplementary Fig. S1B). However, OTUD6B knockdown significantly decreased migration and invasion (Supplementary Fig. S1C–E).

OTUD6B exhibited no significant impact on the proliferative phenotype but enhanced migration and invasion. We selected the CRC liver metastasis model for further verification. We administered OTUD6B-overexpressing or control HCT116 cells into randomized BALB/c nude mice using intrasplenic injection. OTUD6B overexpression significantly enhanced CRLM, manifested by increased tumor nodules and tumor area (Fig. 2F, G). Furthermore, similar results were observed when Otud6b-overexpressing MC38 cells were injected into BALB/c nude mice (Supplementary Fig. S2A, B). These results collectively demonstrated that OTUD6B facilitated CRC cell migration and liver metastasis.

Our data indicated a pro-tumoral role of OTUD6B, which was inconsistent with the previous survivorship curve, indicating that OTUD6B exerts an antitumor effect on colorectal cancer in patients with CRC (Fig. 1G). Consequently, we created a liver metastasis model of CRC by injecting MC38 cells into C57BL/6J mice. Unexpectedly, Otud6b-overexpressing C57BL/6J mice exhibited tumor suppression rather than progression. Otud6b reduced the number of tumor nodules and the tumor area (Fig. 2H, I). Therefore, Otud6b exhibited opposite effects on CRLM in immunodeficient and immunocompetent mice.

### OTUD6B promotes T-cell infiltration to inhibit CRC metastasis in immunocompetent mice

Nude mice lack a proper thymus, resulting in mature T cell deficiency, while C57BL/6J mice are immunocompetent. We hypothesized that host immunological responses, especially T cells, mediate the contradictory effects of OTUD6B. We examined whether OTUD6B was associated with adaptive immune cell profiles and found that OTUD6B was significantly positively correlated with CD8^+^ T cells and negatively correlated with Treg cells on the TIMER database (Supplementary Fig. S3A). Additionally, survival was prolonged in patients with CRC solely when OTUD6B and CD8^+^ T cells were highly expressed. However, OTUD6B expression and Treg exhibited no significant effect on survival time (Supplementary Fig. S3B).

**Fig. 3:**
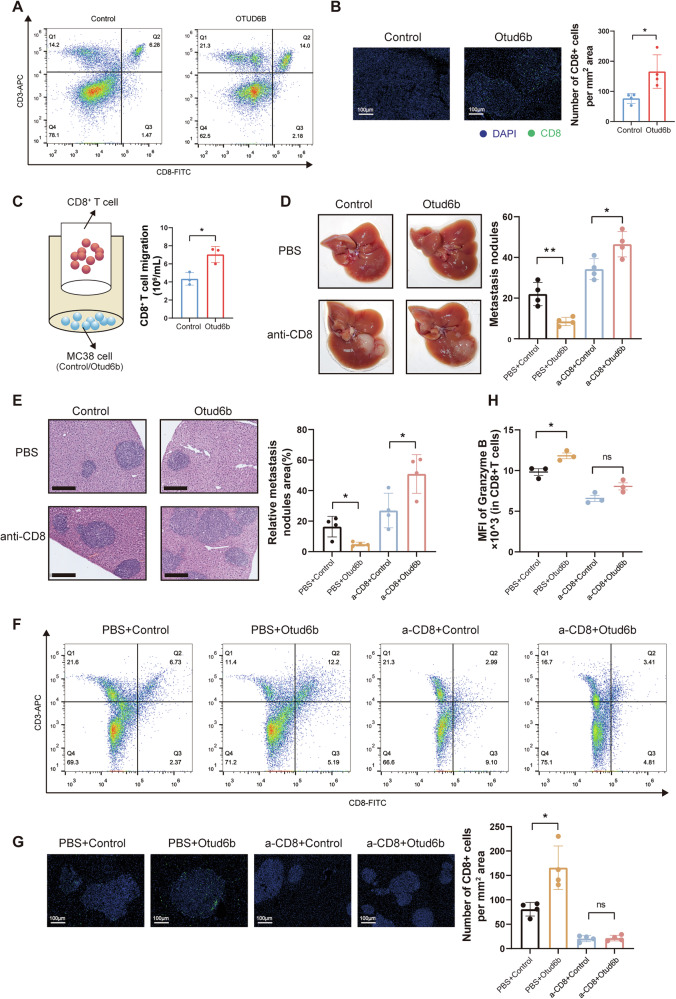
OTUD6B promotes T-cell infiltration to inhibit CRC metastasis in immunocompetent mice. **A** Representative flow cytometry plots and quantification of CD3^+^ CD8^+^ T cells for the indicated groups. **B** Representative IF staining and quantification of CD8^+^ T cells in tumor tissues for the indicated groups (*n* = 4). Scale bar = 100 µm. **C** The schematic diagram of the CD8^+^ T cell migration test (left). Relative migration of mouse CD8^+^ T cells co-incubated with culture medium supernatant from Control or Otud6b-overexpression MC38 cells (right) (*n* = 3). **D** Representative pictures and quantitative results of liver metastatic nodules from C57BL/6J mice intraperitoneally injected with or without anti-CD8 antibody before treatment (*n* = 4). **E** Representative pictures and quantitative results of H&E staining of liver tissue sections from (**D**). **F** Representative flow cytometry plots and quantification of CD3^+^ CD8^+^ T cells for the indicated groups. **G** Representative IF staining and quantification of CD8^+^ T cells in tumor tissues for the indicated groups. Scale bar = 100 μm. **H** The statistical data for Granzyme B^+^ CD8^+^ T cells in the liver cancer tissues. Data are expressed as mean ± SEM. A two-sided Student’s *t* test was used for the statistical analysis. *P < 0.05; ns not significant.

We utilized flow cytometry analysis on C57BL/6J mice to investigate whether CD8^+^ T cells changed in immunocompetent mice and found an increased number of CD8^+^ T cells in tumor tissue of C57BL/6J mice exhibiting high Otud6b expression (Fig. 3A). Immunofluorescence (IF) assay revealed that Otud6b expression in tumor cells was positively correlated with CD8^+^ T cell infiltration in liver metastasis (Fig. 3B). The proportion of CD8^+^ CD69^+^ T cells was significantly increased in mice with high Otud6b expression, indicating that Otud6b might facilitate the early activation of CD8^+^ T cells. Similarly, Otud6b increased the late-stage activation of T cells, manifested by increased CD8^+^ CD44^+^ T cells upon Otud6b overexpression (Supplementary Fig. S4A). Consistent with the increased activated CD8^+^ T cells, the expression of inflammation-related genes, including *Tnfa* and granzyme A (*Gzma*), were higher (Supplementary Fig. S4B). Through an in vitro T cell migration assay, we confirmed that Otud6b overexpression in mouse MC38 cells enhanced CD8^+^ T cell migration (Fig. 3C). Additionally, Otud6b knockdown promoted liver metastasis of CRC in C57BL/6J mice (Supplementary Fig. S5A, B). Besides, Otud6b knockdown inhibited the amount and activation of CD8^+^ T cells in C57BL/6J mice tumor tissue (Supplementary Fig. S5C–E). In an in vitro T cell migration assay, Otud6b knockdown in mouse MC38 cells inhibited CD8^+^ T cell migration (Supplementary Fig. S5F).

To confirm that CD8^+^ T cells mediate the tumor-supressing effects of Otud6b-expressing MC38 cells in C57BL/6J mice, we depleted CD8^+^ T cells using administration of anti-CD8 antibody in the CRC liver metastasis model. CD8^+^ T cell depletion facilitated liver metastasis in CRC in the control and Otud6b-overexpression groups. However, upon depletion of CD8^+^ T cells, the Otud6b overexpression group exhibited an increased number of liver metastasis than the control group, reversing the tumor-suppressive effect of Otud6b in immunocompetent mice (Fig. 3D, E). Flow cytometry and IF assay confirmed a reduction in the number and killing function of CD8^+^ T cells after administering anti-CD8 antibody. The number of CD8^+^ T cells exhibited no significant difference between the control and Otud6b overexpression groups when both groups were treated with anti-CD8 antibodies (Fig. 3F–H). Furthermore, we utilized OT-1 mice, which have CD8^+^ T cells specific for a peptide epitope of the model antigen ovalbumin (OVA), to create a liver metastasis model of CRC. When CD8^+^ T cells were activated, colorectal cancer liver metastasis was suppressed and Otud6b inhibited liver metastasis (Supplementary Fig. S6A–D). Otud6b inhibited CRLM through CD8^+^ T cells in immunocompetent mice.

### OTUD6B promotes CD8^+^ T cell infiltration by upregulating CXCL11

**Fig. 4:**
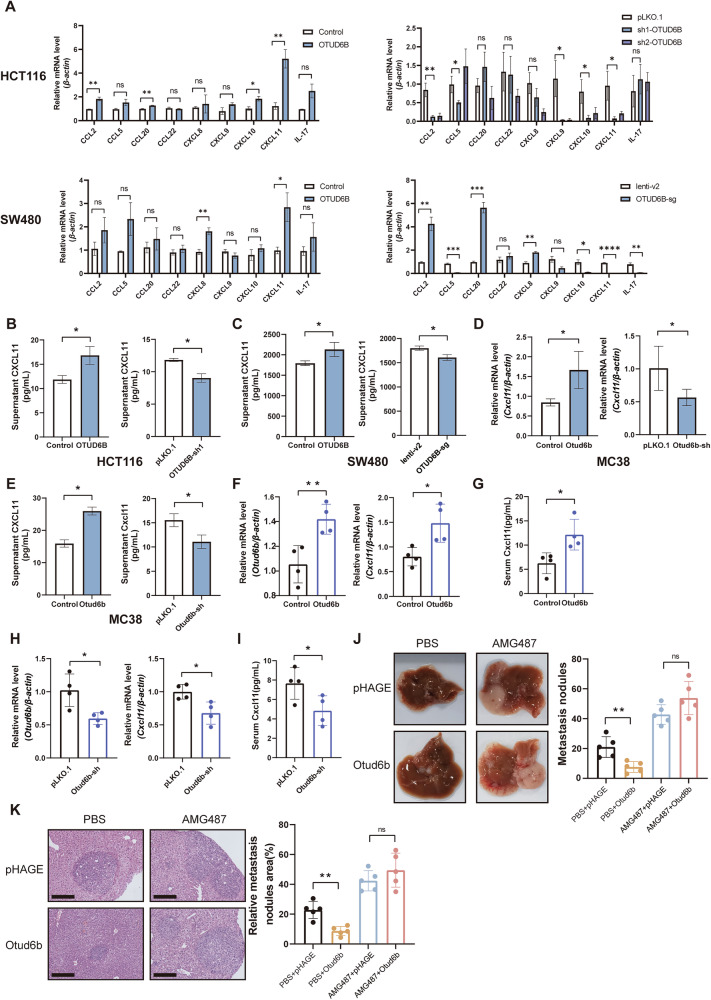
OTUD6B promotes CD8^+^ T cell infiltration by upregulating CXCL11. **A** The relative mRNA levels of chemokines from OTUD6B-overexpression (left) or knockdown (right) HCT116 and SW480 cells. **B**, **C** Cellular supernatant, CXCL11, was detected using an ELISA kit from human CRC cells (HCT116 and SW480). **D** The relative mRNA levels of *Cxcl11* from Otud6b overexpression (left) or knockdown (right) MC38 cells. **E** Cellular supernatant Cxcl11 was detected by ELISA kit from mouse CRC cells (MC38). **F** The relative mRNA levels of *Otud6b* (left) and *Cxcl11* (right) from liver tissues injected with MC38-Otud6b-overexpression cells in C57BL/6J mice (*n* = 4). **G** Serum Cxcl11 was detected using an ELISA kit from (**F**). **H** The relative mRNA levels of *Otud6b* (left) and *Cxcl11* (right) from liver tissues injected with MC38-Otud6b-knockdown cells in C57BL/6J mice (*n* = 4). **I** Serum Cxcl11 was detected using an ELISA kit from (**H**). **J** Representative pictures and quantitative results of liver metastatic nodules from C57BL/6J mice intraperitoneally injected with or without AMG487 (CXCR3 inhibitor) treatment (*n* = 5). **K** Representative pictures and quantitative results of H&E staining of liver tissue sections from (**J**). Data are expressed as mean ± SEM. A two-sided Student’s *t* test was used for the statistical analysis. *P < 0.05; **P < 0.01; ***P < 0.001; ns not significant.

Given the increase in CD8^+^ T cell counts, we analyzed the mRNA level of various chemokines that might recruit CD8^+^ T cells. Among them, *CXCL11* changes were particularly notable. CXCL11 demonstrated a positive correlation with OTUD6B across various human CRC cells (Fig. 4A). The protein level of CXCL11 also changed along with OTUD6B in HCT116 and SW480 by ELISA (Fig. 4B, C). The mRNA and protein changes of Cxcl11 are consistent with Otud6b in murine-derived MC38 cells (Fig. 4D, E). Moreover, Otud6b increased the mRNA expression of *Cxcl11* in the mouse model of liver metastasis of CRC (Fig. 4F). The protein of Cxcl11 also increased when Otud6b was overexpressed (Fig. 4G). Besides, the Otud6b knockdown exhibited the opposite effect (Fig. 4H, I). OTUD6B upregulated CXCL11 in CRC liver metastasis.

Furthermore, CXCR3 (the receptor of CXCL11) knockdown reverted the increased migration because of OTUD6B overexpression in HCT116 cells (Supplementary Fig. S7A–C). The CXCR3 inhibitor AMG487 promoted CRLM and reversed the antitumor effect of Otud6b in C57BL/6J mice (Fig. 4J, K and Supplementary Fig. S7D). OTUD6B inhibited tumor metastasis through CXCL11-CXCR3 axis.

### OTUD6B stabilizes DDX5 protein expression

**Fig. 5:**
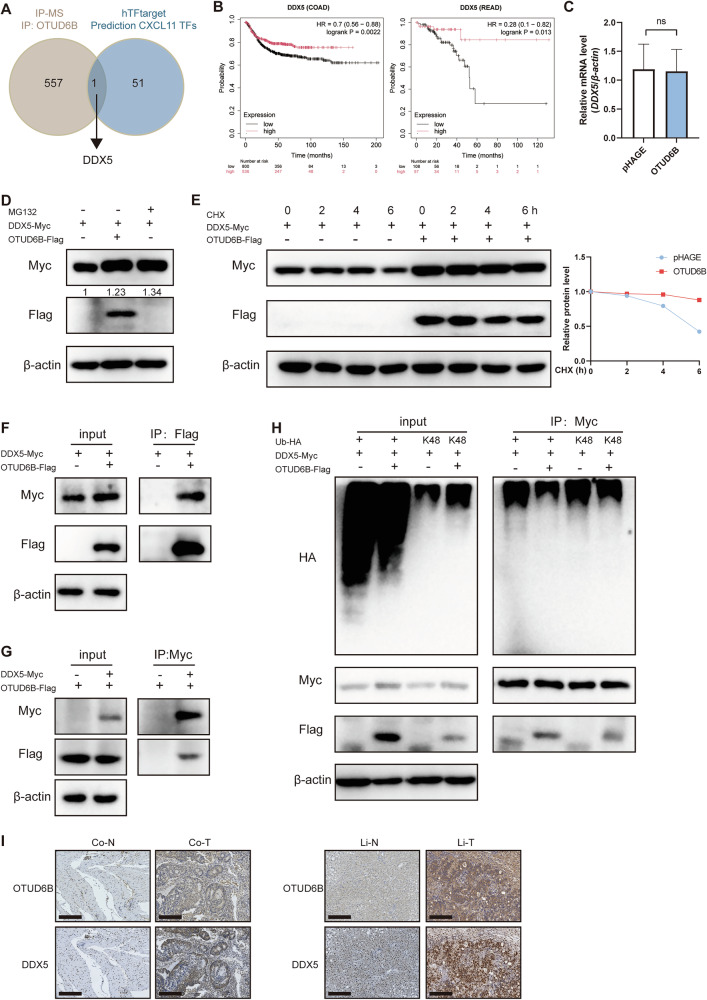
OTUD6B stabilizes DDX5 protein expression. **A** There were 558 proteins interacting with OTUD6B in HCT116 cells through IP-MS and 52 possible transcription factors to transcribe CXCL11 from hTFtarget. Take the intersection of IP-MS and transcription factor prediction. **B** Survival analysis between patients with DDX5 high- and low-expression COAD and READ using kmplot database. **C** The relative mRNA level of *DDX5* from OTUD6B-overexpression or control HCT116 cells. **D** HCT116 cells were transfected with the indicated plasmids, followed by treatment with MG132 (5 µM) or not for 12 h before collection. **E** HCT116 cells were transfected with the indicated plasmids for 48 h. Cells were subsequently treated with cycloheximide (CHX, 50 µM) and collected for immunoblot analysis at the indicated time points. **F**, **G** HCT116 cells transfected with indicated plasmids for 48 h. Cells were lysed with IP buffer and subsequently analyzed using co-IP with Flag or Myc antibody followed by Western blot. **H** HCT116 cells were transfected with the indicated plasmids, followed by treatment with MG132 (5 µM) for 12 h before collection. The lysates were incubated with Myc antibody and then subjected to immunoblotting. **I** Representative images of IHC staining of OTUD6B and DDX5 expression in colorectal tumor (Co-T) and adjacent normal (Co-N) specimens (left). Representative images of IHC staining of OTUD6B and DDX5 expression in liver tumor (Li-T) and adjacent normal (Li-N) specimens (right). Scale bar = 200 µm. Data are expressed as mean ± SEM. A two-sided Student’s t-test was used for the statistical analysis. ns, not significant.

Otud6b inhibited CRLM through Cxcl11 in immunocompetent mice. The deubiquitinase OTUD6B removed ubiquitination from its substrates and regulated the stability and functions of its substrates. We identified 558 proteins that interact with OTUD6B using immunoprecipitation and mass spectrometry (IP-MS) to screen the potential substrate proteins of OTUD6B. At the same time, we predicted CXCL11 transcription factors within the hTFtarget database. The intersection of IP-MS and hTFtarget revealed the unique molecule DDX5 (Fig. 5A). High DDX5 expression in CRC samples correlated with longer survival in the kmplot database, alongside OTUD6B (Fig. 5B). Ubiquitin is a type of post-translational modification; alterations in protein level in response to DUB overexpression or interference did not induce changes in transcription. As expected, the *DDX5* mRNA level remained unchanged with OTUD6B overexpression (Fig. 5C). We subsequently treated HCT116 with ectopic expression of OTUD6B or with a proteasome inhibitor (MG132) to understand the regulatory effect of OTUD6B on DDX5 protein. OTUD6B overexpression and MG132 significantly increased DDX5 protein level, indicating that OTUD6B stabilized DDX5 through the ubiquitin-proteasome pathway (Fig. 5D). We subsequently assessed the DDX5 protein level in the presence of CHX, a protein translation inhibitor. Notably, OTUD6B overexpression resulted in a significant increase in DDX5 stability (Fig. 5E). Subsequently, we investigated the interaction between OTUD6B and DDX5 by co-immunoprecipitation (co-IP) to verify the association of DDX5-Myc with OTUD6B-Flag. Consistent with our expectations, we observed that OTUD6B-Flag interacted with DDX5-Myc (Fig. 5F, G). Given that OTUD6B is a DUB, we examined whether OTUD6B inhibited DDX5 ubiquitylation. The ectopic expression of OTUD6B inhibited DDX5 ubiquitylation and Lys 48-linked ubiquitylation (K48, primarily responsible for protein degradation) in HCT116 cells (Fig. 5H). Furthermore, we investigated other types of ubiquitin chain of DDX5 affected by OTUD6B, which also diminished K33 ubiquitylation (Supplementary Fig. S8A). We confirmed that OTUD6B was positively correlated with DDX5 in human CRC and liver metastasis samples (Fig. 5I). Collectively, OTUD6B deubiquitinated and stabilized DDX5.

We simultaneously overexpressed Otud6b and knocked down Ddx5 to investigate whether Ddx5 is a downstream molecule of Otud6b. Ddx5 knockdown promoted CRC liver metastasis and eliminated the effect of Otud6b (Supplementary Fig. S9A–C). It demonstrated that OTUD6B inhibited liver metastasis of CRC by stabilizing DDX5.

### DDX5 promotes STAT3 mRNA translation and CXCL11 transcription by resolving the rG4 structure of STAT3

**Fig. 6:**
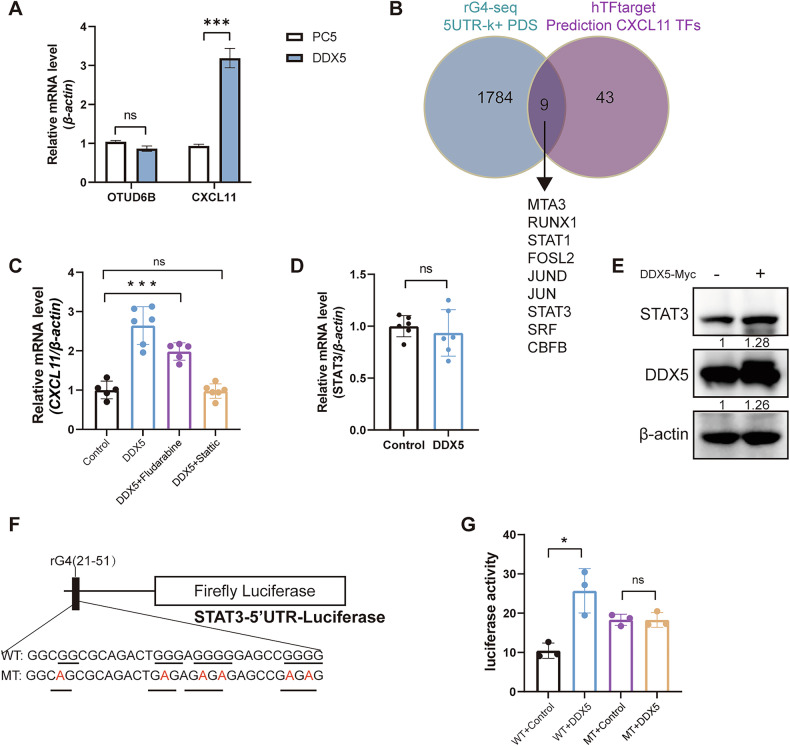
DDX5 promotes STAT3 mRNA translation and CXCL11 transcription by resolving the rG4 structure of STAT3. **A** The mRNA levels of *OTUD6B* and *CXCL11* from DDX5-overexpression HCT116 cells. **B** The intersection of rG4 sequencing and transcription factor prediction. Sequencing revealed 1793 genes with rG4 in 5’UTR and 52 transcription factors transcribed CXCL11 from hTFtarget. **C** The mRNA level of *CXCL11* from DDX5-overexpression HCT116 cells after treatment with 5 µM STAT1 inhibitor (fludarabine) or STAT3 inhibitor (stattic) for 24 h. **D** The mRNA level of *STAT3* from control and DDX5-overexpression HCT116 cells. **E** HCT116 cells transfected with vector or DDX5 for 48 h. **F** Putative WT rG4 sequences in the human STAT3 5’UTR nucleotide sequence are indicated. The red fonts indicate site-directed changes in mutant MT-rG4. **G** Firefly/Renilla luciferase activity ratio at 24 h after co-transfection of WT or MT STAT3-5’UTR. Data are expressed as mean ± SEM. A two-sided Student’s *t* test was used for the statistical analysis. *P < 0.05; ***P < 0.0001; ns not significant.

DDX5 overexpression increased the mRNA level of *CXCL11* without affecting *OTUD6B* (Fig. 6A). RNA helicase DDX5 could regulate transcription factor STAT1 mRNA translation by resolving an RNA G-quadruplex (rG4) structure proximal to the 5’ end of STAT1 5′UTR [13], rather than functioning directly as a transcription factor. Kwok et al. identified 1793 mRNA 5’UTR in humans that might contain rG4 structures by rG4-seq transcriptomic studies [18]. We aimed to identify a transcription factor regulated by DDX5 that simultaneously transcribes CXCL11. Therefore, we intersected the rG4-seq transcriptomic analysis with the CXCL11 transcription factors predicted by the hTFtarget database, resulting in the identification of nine transcription factors (Fig. 6B). We examined the correlation between the expression of these transcription factors and CRC survival using kmplot and protein atlas databases. Only the expression of STAT1 and STAT3 were positively correlated with the survival time in both databases (Supplementary Fig. S10A–C). To determine which transcription factor regulated *CXCL11* expression, we transfected the DDX5 overexpression plasmid and administered STAT1 or STAT3 inhibitors to measure *CXCL11* mRNA level in HCT116 cells. *CXCL11* was upregulated because of DDX5 overexpression and was reversed by treatment with stattic (STAT3 inhibitor), not fludarabine (STAT1 inhibitor) (Fig. 6C). *STAT3* mRNA level was unaffected in DDX5-overexpressing HCT116 cells compared to control cells (Fig. 6D), thereby excluding DDX5 effects on STAT3 transcription. Subsequently, we transfected DDX5 into HCT116 cells. The increase in DDX5 protein increased STAT3 protein level (Fig. 6E). We constructed a mutant (MT) rG4, with G to A substitutions within the putative rG4 sequence, to investigate whether DDX5 regulation on STAT3 was mediated by rG4 structure (Fig. 6F). HCT116 cells subjected to control or DDX5 overexpression were simultaneously transfected with WT or MT rG4, resulting in a significant increase in F. luciferase activity from MT rG4 in comparison with the WT rG4 vector in the control group. And DDX5 increased F. luciferase activity only from the WT rG4 containing vector, without an effect on MT rG4 (Fig. 6G). Collectively, these results identify the rG4 sequence as a functional element in the 5’UTR of STAT3 mRNA, regulating its translation.

### ATRA upregulates OTUD6B to inhibit CRC liver metastasis

**Fig. 7:**
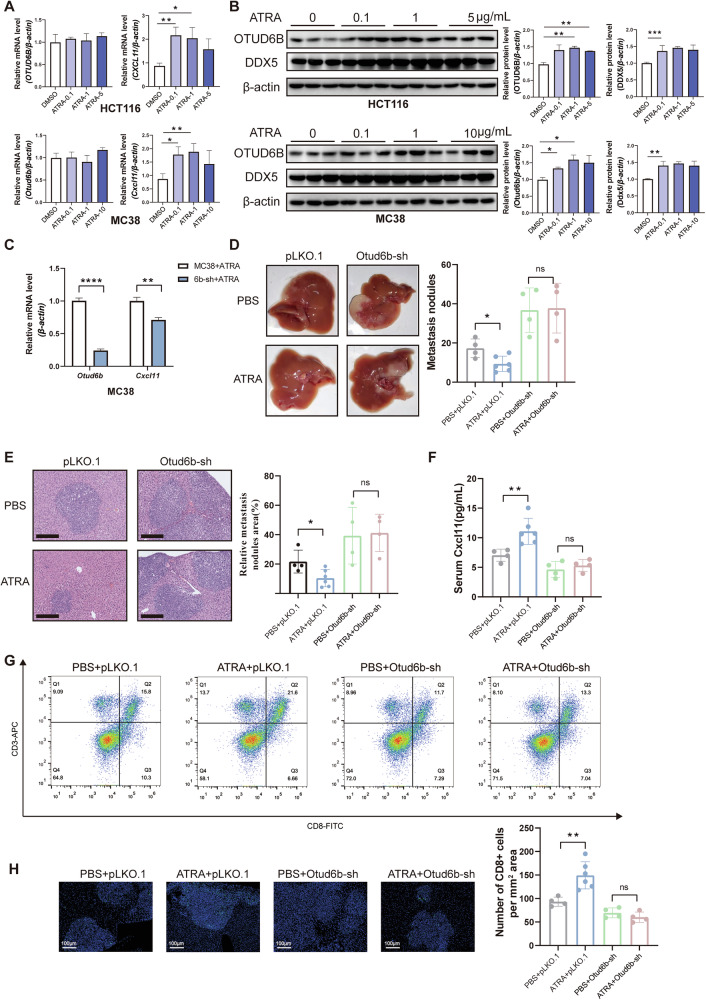
ATRA upregulates OTUD6B to inhibit CRC liver metastasis. **A** The mRNA levels of *OTUD6B* and *CXCL11* in HCT116 or MC38 cells treated with ATRA in the indicated doses for 48 h. **B** The protein levels of OTUD6B and DDX5 in HCT116 or MC38 cells treated with ATRA in the indicated doses for 48 h. **C** The mRNA levels of *Otud6b* and *Cxcl11* from control or *Otud6b*-knockdown MC38 cells treated with ATRA (1 µg/mL) for 48 h. **D** Representative pictures and quantitative results of liver metastatic nodules from C57BL/6J mice intraperitoneally injected with or without ATRA treatment (*n* = 4–6). **E** Representative pictures and quantitative results of H&E staining of liver tissue sections from (**D**). **F** Serum Cxcl11 detected using an ELISA kit from (**D**). **G** Representative flow cytometry plots and quantification of CD3^+^ CD8^+^ T cells for the indicated groups. **H** Representative IF staining and quantification of CD8^+^ T cells in tumor tissues for the indicated groups. Scale bar = 100 µm. Data are expressed as mean ± SEM. A two-sided Student’s *t* test was used for the statistical analysis. *P < 0.05; **P < 0.01; ****P < 0.0001; ns not significant.

All-trans retinoic acid (ATRA) enhanced OTUD6B protein level, hence inhibiting esophageal squamous cell carcinoma (ESCC) progression [8]. We attempted to evaluate OTUD6B mRNA and protein expression in HCT116 and MC38 cells after treatment with various concentrations of ATRA. The mRNA level exhibited no significant difference; however, the protein level was upregulated upon ATRA treatment (Fig. 7A, B). Additionally, the mRNA level of *CXCL11* was upregulated following ATRA treatment (Fig. 7A), and the protein level of DDX5 was similarly upregulated after ATRA treatment (Fig. 7B). We treated Otud6b-knockdown cells with ATRA to investigate whether ATRA regulated *Cxcl11* through Otud6b and found that *Cxcl11* still reduced (Fig. 7C). It showed that ATRA regulated CXCL11 through OTUD6B. Furthermore, ATRA inhibited liver metastasis of CRC in C57BL/6J mice, while OTUD6B-knockdown exhibited a diminished response to ATRA treatment (Fig. 7D, E). The protein level of CXCL11 was upregulated after ATRA treatment in the pLKO.1 group (Fig. 7F). Flow cytometry and IF assays verified that the number of CD8^+^ T cells upregulated after ATRA administration in the pLKO.1 group. Moreover, CD8^+^ T cells exhibited no significant difference between PBS and ATRA groups when both groups underwent Otud6b knockdown (Fig. 7G, H). Collectively, these results revealed the potential that ATRA might enhance the protein level of OTUD6B for treating CRLM.

## Discussion

CRC is the third most common cancer, and the liver is the most common organ for CRC metastasis. However, studies on the deubiquitinase OTUD6B in CRC liver metastasis are rare. This study demonstrated that the expression of OTUD6B was upregulated in colorectal clinical samples and cell lines. OTUD6B facilitated the metastasis of CRC cells and CRC in nude mice, while it inhibited CRC liver metastasis in C57BL/6J mice. Accordingly, we provided evidence to demonstrate the functional role of OTUD6B in recruiting CD8^+^ T cell infiltration to inhibit liver metastasis in immunocompetent mice, including C57BL/6J. Additionally, we revealed a mechanism by which OTUD6B stabilized the DDX5-STAT3-CXCL11 signaling axis and recruited CD8^+^ T cells to enhance the immune response in liver metastasis of CRC. Targeting OTUD6B with ATRA yielded an enhanced antitumor effect, indicating a potential therapeutic strategy for CRC liver metastasis.

Pan-cancer analysis revealed that high *OTUD6B* mRNA expression was observed in 10 tumors, including COAD and READ [19]. High OTUD6B expression resulted in an adverse outcome of multiple myeloma [20]. Furthermore, OTUD6B facilitated tumor progression in many cancers, including lung adenocarcinoma, laryngeal squamous cell carcinoma, triple-negative breast cancer, and cholangiocarcinoma [10, 21–23]. But OTUD6B inhibited hepatocellular carcinoma metastasis and suppressed cell migration in clear cell renal cell carcinoma [6, 24]. These studies were conducted in cellular and immunodeficient mice, specifically nude mice and NOD SCID mice.

OTUD6B suppressed ESCC in NOD/SCID mice (immunodeficient) and C57BL/6N mice (immunocompetent). Furthermore, ATRA facilitated OTUD6B ptotein expression, and its combination enhanced the responsiveness of ESCC tumors to anti-PD-1 immunotherapies [8]. However, the investigation did not examine the role of OTUD6B in tumor immunity. Mao’s study identified six autoantibodies, including OTUD6B, were present in mice prior to the development of mammary cancer through spontaneous mammary tumor models in transgenic mice (TgMMTV-neu). it was useful for the early detection of human breast cancer [25]. Furthermore, in mouse mammary tumors, vaccination with tumor antigens (Otud6b, Pdhx or Stk39) induced tumor-specific CD4^+^ and CD8^+^ T cells, along with tumor-specific IFN-g T cell and CD8^+^ T cell responses. The type I antigen-specific T-cell immune response was responsible for inhibiting tumor growth. The depletion of T cells, rather than B cells, abrogated the growth inhibition resulting from vaccination with the early-stage antigens. It suggested that antigen (including OTUD6B) expressed earlier in breast tumor development might be effective targets for therapeutic breast cancer vaccines [26]. Consistent with our study, OTUD6B inhibited tumors by activating CD8^+^ T cell responses.

Our study demonstrated that OTUD6B exhibited contrasting roles in different immune conditions. OTUD6B facilitated CRLM in immunodeficient mice. However, it inhibited CRLM in immunocompetent mice. This may be attributable to the existence of dual secretory mechanisms of CXCL9/10/11-CXCR3 axis. It modulates immune cell migration, differentiation, and activation, resulting in tumor suppression (paracrine axis). However, the previous study demonstrated the involvement of this axis in tumor growth and metastasis (autocrine axis) [27]. Another previous study has demonstrated that CXCR3 activation with its ligands stimulates colon cancer metastasis [28]. However, Torphy’s study demonstrated that CXCR3 blockade reverses the improved antitumor immunity and T-cell infiltration observed in GPR182-deficient mice [29]. Our study demonstrated similar results, CXCR3 knockdowns inhibited CRC cell migration, while CXCR3 inhibitors diminished CD8^+^ T cell infiltration and facilitated tumor metastasis. CXCR3 expression is mainly expressed in activated T cells and some epithelial and endothelial cells [30].

The liver is the most common organ for distant metastasis in CRC [31]. Blood from the gastrointestinal tract flows into the liver through the portal vein, facilitating the dissemination of CRC into the liver [32, 33]. The tumor microenvironment (TME) is crucial in CRLM. TME of liver metastasis exhibits a highly immunosuppressive phenotype, signifying a depletion of antigen-specific CD8^+^ T cells. Consequently, this TME enhances the invasion and metastasis capabilities of the primary cancer cells [34, 35]. Blocking arachidonic acid metabolism could enhance immune responses against tumors by activating CD8^+^ T cells in CRC [36]. Tumor-associated macrophages induced apoptosis of CD8^+^ T cells and impair cytotoxic functions by reducing granzyme B and perforin expression in the liver [37]. MGP could facilitate CD8^+^ T cell exhaustion by activating the NF-κB pathway; the combination of MGP knockdown and αPD1 could synergistically resist liver metastasis of CRC [38]. These studies highlight the need to reactivate CD8^+^ T cells to inhibit the advancement and metastasis of CRC.

The therapeutic targeting of immune checkpoint molecules is crucial in tumor therapy. We analyzed the mRNA expression of *Pd-l1* and *Ctla4* in liver tissues of Otud6b-overexpressed and control mice with CRLM, revealing a decrease solely in *Pd-l1*. Similarly, Pd-l1 protein expression was decreased (data not shown). Otud6b also inhibited the number of CD8^+^ T cells exhibiting high Pd-1 expression (data not shown). We hypothesized that the recruitment of CD8^+^ T cells and the decrease of PD-L1 expression in tumor cells resulted in a decline of depleted CD8^+^ T cells and increased active CD8^+^ T cells, potentially enhancing the tumoricidal efficacy of CD8^+^ T cells. The specific mechanism through which OTUD6B regulates PD-1/PD-L1 requires more investigation.

From a therapeutic perspective, it is difficult to recover CD8^+^ T cell infiltration by directly rescuing the expression of STAT3 or CXCL11. Utilizing ATRA to upregulate OTUD6B and subsequently stabilize the expression of its downstream DDX5, we hypothesized that targeting OTUD6B protein expression is a viable technique for combating cancer. ATRA is efficacious against acute promyelocytic leukemia [39]. ATRA was gradually being utilized in many solid tumors, including CRC [8, 40, 41]. An unprecedented correlation exists between immune system activation and ATRA supplementation, while one of the immune-related mechanisms indicates that the benefits of ATRA treatment are mediated by cytotoxic CD8^+^ T cells, stimulated due to MHCI upregulation on tumor cells [42]. The mechanism of ATRA in tumor immunity requires additional investigation.

In conclusion, this study demonstrated that OTUD6B exhibited contrasting effects in immunodeficient and immunocompetent mice. OTUD6B recruited CD8^+^ T cells to inhibit CRC liver metastasis through the DDX5-STAT3-CXCL11 axis. Besides, we considered ATRA as an agonist of OTUD6B to inhibit CRC liver metastasis.

## Materials and methods

### Human primary samples

We acquired nine human CRC and liver metastasis with paired non-tumor samples and clinical information from patients at Wuhan Union Hospital. The patients signed informed consent forms before participating in this study. Samples were immediately frozen in liquid nitrogen and transferred to −80 °C for long-term storage. Human studies were approved by the Ethics Committee of Wuhan Union Hospital (UHCT-IEC-SOP-007-02-05) and followed the principles of the Declarations of Helsinki and Istanbul.

### Animals

We procured C57BL/6J (male, 6–8 weeks old) and BALB/c nude mice (male, 4–6 weeks old) from Beijing Vital River Laboratory (Beijing, China), and OT-1 mice (male, 6–8 weeks old) were obtained from Shulb (Wuhan, China). Mice were reared under special-pathogen-free (SPF) conditions at the Experimental Animal Center of Huazhong University of Science and Technology. All animal-related procedures and experiments were approved by the Institution Animal Use and Care Committee of Huazhong University of Science and Technology (IACUC Number: 4236).

### Cell lines

HCT116, SW480, SW620, MC38, and HEK293T cells were obtained from the American Type Culture Collection (ATCC). CRC cells were cultured in RPMI-1640 medium (gibco, 21875034, California, USA) while HEK293T cells were maintained in DMEM (C3113-0500, Biological Industries) at 37 °C in 5% CO_2_. The medium was supplemented with 10% fetal bovine serum (gibco, A3160902, California, USA) and 1% penicillin/streptomycin (Invitrogen, 15140163, California, USA). All experiments were conducted using cells within 15 passages post-thawing. All cell lines were confirmed as mycoplasma-negative using the PCR method.

### Immunohistochemical (IHC) and immunofluorescence (IF) staining

Paraffin-embedded colorectal and liver sections were dewaxed in xylene and rehydrated using graded alcohol. After heat-induced epitope retrieval in 10 mM citrate buffer, sections were stained with primary antibodies as specified in Supplementary Table S1 and visualized using DAB/AEC or fluorescent-labeled secondary antibodies.

### Mouse models for liver metastasis induction

Liver metastasis was induced by injecting cancer cells intrasplenically in mice. While under anesthesia, the spleen was exposed, and subsequently, CRC cells (2 × 10^6^ HCT116 or 5 × 10^5^ MC38) dissolved in 100 µL PBS were intrasplenically injected into the mice. After 6 weeks, the BALB/c nude mice (C57BL/6J and OT-1 mice, after 2 weeks) were euthanized, and their livers were harvested for further analysis.

For the CD8^+^ T cell deletion assay, 5 × 10^5^ MC38 cells were intrasplenically inoculated into C57BL/6J mice. Anti-CD8 antibodies (200 µg/per mouse) were intraperitoneally administered every 4 days, commencing from the day before the inoculation and continuing until the endpoint.

In the AMG487 treatment assay, 5 × 10^5^ MC38 cells were intrasplenically inoculated into C57BL/6J mice. AMG487 (5 mg/kg) was intraperitoneally administered every 2 days from the day before the inoculation until the endpoint.

For the ATRA treatment assay, 5 × 10^5^ MC38 cells were intrasplenically inoculated into C57BL/6J mice. ATRA (5 mg/kg) was intraperitoneally administered every 4 days from the inoculation until the endpoint.

### Hematoxylin and eosin (H&E) staining

Livers were fixed in 4% paraformaldehyde overnight at 4 °C and embedded in paraffin after dehydration in ascending concentrations of ethanol for histopathological examination of liver metastasis. Sections of each sample were prepared at a thickness of 2 mm at three different levels and stained using H&E). Sections were examined and imaged using the Mshot microscope.

### Flow cytometry

We obtained the liver cancer tissue samples from the respective groups treated with the MC38 cell line. We utilized the mouse tumor dissociation kit (absin, Shanghai, China) to process tumor tissues from mice, whereby Percoll eliminates debris and lyses red blood cells. Subsequently, the cells were washed and stained with fluorochrome-conjugated antibodies (Supplementary Table S1). The data were collected using flow cytometry (Beckman Coulter, Fullerton, CA, USA) and analyzed using FlowJo software (TreeStar, Ashland, OR, USA).

### Lentiviral transduction

HEK293T cells cultured in a 10 cm dish were co-transfected with 6 µg psPAX2, 4 µg pMD2.G, and 8 µg pHAGE-OTUD6B or pLKO.1-OTUD6B-sh to produce lentiviral particles. The medium was collected and filtered with a 0.45 µm filter (Biosharp, BS-PES-45, Hefei, China) 60 h after transfection. Viral supernatant mixed with 8 µg/mL polybrene was utilized to infect the target cells for 6 h and was subsequently replaced with fresh medium. Stable cell lines were selected using 1 µg/mL puromycin treatment 48 h after transfection. The OTUD6B protein levels in various cells were assessed using Western blot analysis.

### CRISPR/Cas9 knockout

We designed single guide RNAs (sgRNAs) utilizing the CRISPR tool (http://crispr.mit.edu) and cloned them into the LentiCRISRPv2 vector, thereafter co-transfected with packaging vectors to HEK293T cells to generate lentivirus. Knockout clones were generated by transducing SW480 cells with lentivirus, and after 48 h, cells were selected with puromycin at 1 µg/mL for 1–2 weeks to obtain stable cells.

### Cell proliferation and viability assays

HCT116 or SW480 cells were planted at 1 × 10^3^/well into 96-well plates. Cell Counting Kit-8 solution (Abclonal, RM02823, Wuhan, China) was added to each well simultaneously for 6 consecutive days, and the OD value was determined by measuring the absorbance at 450 nm.

### Wound-healing assay

HCT116 or SW480 cells were inoculated into 12-well plates with 2 × 10^5^ per well, respectively. The confluent cell monolayer was damaged by scraping the cells with a 1 mL pipette tip at a consistent strength and angle. The images were captured when the cell density reached 80%. Subsequently, the photos were taken at the same time every day for 2 days. Cell migration was evaluated by measuring the differences in the areas of the wounds.

### Transwell migration and invasion assays

The migration and invasion assays were performed as previously described [17]. Cells were collected and resuspended in serum-free RPMI-1640 medium, and subsequently, 5 × 10^5^ HCT116 or SW480 cells were seeded in the upper chamber (Corning, 3398, New York, USA) with (invasion) or without (migration) Matrigel (Sigma-Aldrich, E1270, Darmstadt, Germany). The chamber was inserted into a 24-well plate. The lower chamber was filled with RPMI-1640 complete medium. After 24 h incubation, cells in the upper chambers were eliminated using a cotton swab, while cells attached to the lower membrane surface were fixed with 4% paraformaldehyde and stained with 0.1% crystal violet. Cells were photographed using an optical microscope and counted using Adobe Photoshop CS6 software.

### T-cell migration assay

For mouse T cell migration assays, CD8^+^ T cell isolation was conducted as described previously [43]. Splenic T cells were isolated using a CD8^+^ T cell isolation kit and subsequently collected for the co-culture. Additionally, 5 × 10^5^/100 µL of the CD8^+^ T cells were introduced into the transwell insert (5 µm pore size) to allow access to the lower compartment containing the supernatant culture medium from various tumor cells. The cells were cultured in RPMI-1640 medium for 24 h, after which the cells that migrated from the upper compartment to the lower compartment were counted.

### Western blot

We inoculated 1 × 10^6^ cells into a 6-well plate. After 12 h or when the cell density reached 60–80%, plasmids were transfected as indicated. Proteins were taken from whole-cell lysates using RIPA buffer (Beyotime, P0013B, Shanghai, China) supplemented with 1% PMSF (Beyotime, ST506, Shanghai, China) 48 h after transfection, and the protein concentration was quantified using a BCA kit (Aidlab, PP0102, Beijing, China). Supplementary Table S1 presents the primary antibodies.

### Immunoprecipitation (IP) assay

We seeded 3 × 10^6^ HCT116 cells into a 6-cm dish. After 12 h or when the cell density reached 60%–80%, plasmids were transfected as indicated. Cells were lysed using IP buffer (20 mM Tris, 150 mM NaCl, 1 mM EDTA, 1% Triton X-100) supplemented with protease inhibitors, combined with Flag or Myc antibody and agarose beads (Santa Cruz, sc-2336, Texas, USA) 48 h after transfection, and incubated overnight at 4 °C. The immunoprecipitated proteins were eluted by boiling in a loading buffer (250 mM Tris-HCl, 0.1 g/mL SDS, 50% glycerinum, 5 mg/mL bromophenol blue). IP samples and whole-cell lysates were subjected to Western blot assay.

### Reverse transcription-quantitative PCR (qPCR)

Total RNA was isolated with Trizol reagent and subsequently converted into cDNA using the PrimeScript RT Reagent Kit (Vazyme, R323-01, Nanjing, China). Quantitative polymerase chain reaction was conducted using Champagne Taq DNA Polymerase (Vazyme, Q311-02, Nanjing, China) according to the manufacturer’s instructions. ACTB served as an internal control. Relative expression was examined using the 2^−ΔΔCT^ method. Supplementary Table S2 presents the primers used in this study.

### Luciferase reporter assay

The luciferase reporter assays were conducted as previously described [44]. The wild-type or mutated STAT3-5’UTR was cloned into the pGL3-basic vector. HCT116 cells were co-transfected with the specified plasmids. Subsequently, cells were lysed (Cat: E1910; Promega) and detected with the fluorescence detector (Promega GloMax 20/20, USA) according to the manufacturer’s instructions.

### Public dataset-based bioinformatic analysis

We analyzed the differentially expressed genes in CRC liver metastasis and normal tissue using GEO2R from the GEO Datasets (https://www.ncbi.nlm.nih.gov/geo/). The UALCAN database (http://ualcan.path.uab.edu) collected RNA-seq and clinical data of cancer from the TCGA database, providing a valuable platform for the analysis of OTUD6B expression in tumor and normal tissues. The prognostic values of OTUD6B specifically expressed in colon and rectal cancer samples were assessed using overall survival utilizing the Kaplan–Meier plotter resource (http://www.kmplot.com). Besides, we analyzed the correlation between OTUD6B and adaptive immune cell profiles and the effects of OTUD6B and T cells as two variables on survival time in patients with CRC using the TIMER database (http://timer.comp-genomics.org/).

### Statistics and reproducibility

No statistical analysis was employed to determine sample sizes. Sample sizes were established according to previous studies or our experimental experience. The mice were randomized into various drug treatment groups for in vivo experiments. No data were excluded from the analysis. Statistical tests were selected based on the characteristics of the variables and the assumption of data distribution. Data are expressed as mean ± standard error of the mean (SEM). For the liver metastasis model, the number of mice in each group was indicated in each specific experiment. All statistical analyses were performed using GraphPad Prism 7. A two-sided Student’s *t* test was utilized for comparisons between two groups, while a one-way analysis of variance was utilized for multiple comparisons. Specific tests were described in the respective figure legends. *P* < 0.05 was considered statistically significant, with *P < 0.05; **P < 0.01; ***P < 0.001; ****P < 0.0001; ns not significant.

## Supplementary information

Supplementary materials

Original Data

## Data Availability

The CRC dataset analyzed in this study was obtained from GEO at GSE14095, GSE38174 and GSE81582. All other raw data generated in this study are available upon request from the corresponding author.
