# Supplementary material for: ATRA upregulates OTUD6B to recruit CD8+ T cells to suppress colorectal liver metastasis by stabilizing DDX5/STAT3/CXCL11 axis
Source: Cell Death Dis. 2025 Jul 12;16(1):521. doi: 10.1038/s41419-025-07837-0 (PMC12255723; doi:10.1038/s41419-025-07837-0)
Supplement: Supplementary file 1 — Supplementary materials [file 41419_2025_7837_MOESM1_ESM.doc]

**ATRA upregulates OTUD6B to recruit CD8^+^ T cells** **to suppress colorectal liver metastasis by stabilizing DDX5/STAT3/CXCL11 axis**

Jinglei Li^1^, Kunpeng Huang^2^, Bing Yang^1^, Xia hu^1^, Bosheng Mei^1^, Xiang Cheng^3^, Xin Zhong^1^, Chuyi Cao^1^, Zihan Chen^4^, Hui Wang^4^,*, Jinxiang Zhang^1^,*

* Corresponding authors

Addresses: 1277 Jiefang Road, Wuhan, Hubei Province 430022, China (Jinxiang Zhang), or 13 Hangkong Road, Wuhan, Hubei Province 430030, China (Hui Wang).

E-mail address: zhangjinxiang@hust.edu.cn (Jinxiang Zhang), wanghuipitt@hust.edu.cn (Hui Wang)

Table of contents

[Fig. S1 3](#__RefHeading___Toc127037137)

[Fig. S2 5](#__RefHeading___Toc127037138)

[Fig. S3 6](#__RefHeading___Toc127037139)

[Fig. S4 7](#__RefHeading___Toc127037140)

[Fig. S5 8](#__RefHeading___Toc127037141)

[Fig. S6 10](#__RefHeading___Toc127037142)

[Fig. S7 12](#__RefHeading___Toc127037143)

[Fig. S8 13](#__RefHeading___Toc127037144)

[Fig. S9 14](#__RefHeading___Toc127037143)

[Fig. S10 15](#__RefHeading___Toc127037144)

[Supplemental Table1. 17](#__RefHeading___Toc127037145)

[Supplemental Table2. 18](#__RefHeading___Toc127037146)


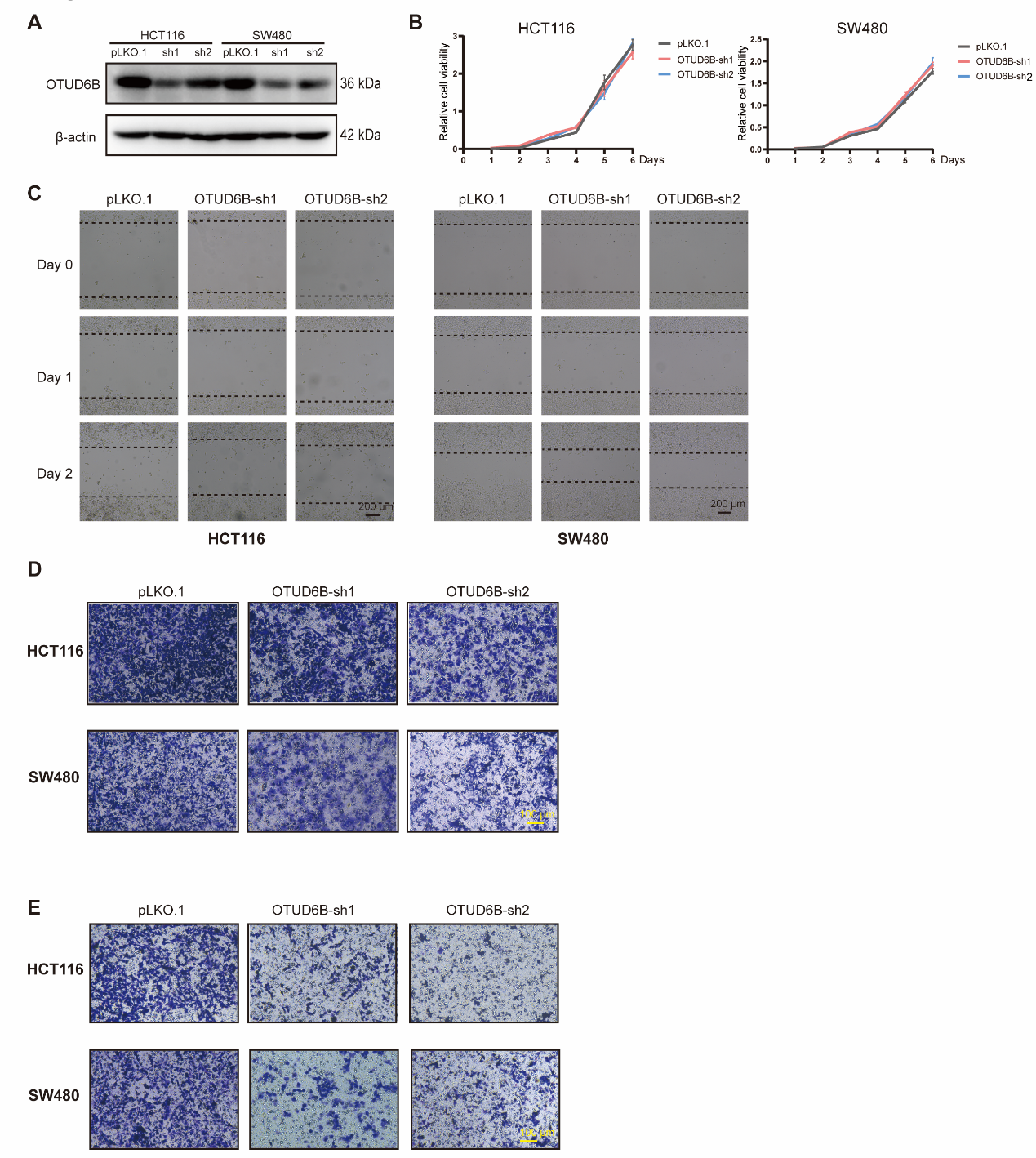


**Figure S1.**

OTUD6B knockdown inhibits migration in CRC cells. **A,** OTUD6B knockdown levels in stable OTUD6B-sh cell lines (HCT116 and SW480) and the corresponding control using Western blot analysis. **B,** Effects of OTUD6B knockdown on the viability of HCT116 and SW480 CRC cells. Cell viability was monitored for 6 days using CCK-8 assay. **C,** Effects of OTUD6B knockdown on the migration of HCT116 and SW480 colorectal cancer cells using a wound-healing assay. Wound closure was determined at 1- and 2-d time points. Scale bar, 200 μm. **D，** Effects of OTUD6B knockdown on cell migration. Control or OTUD6B-sh cells were assayed in Transwell chambers. Migrative potential was assessed after a 24-h incubation. Scale bar, 100 μm. **E,** Effects of OTUD6B knockdown on cell invasion. Control or OTUD6B-sh cells were assayed in Transwell chambers with matrigel. Invasive potential was assessed after a 24-h incubation. Scale bar, 100 μm.


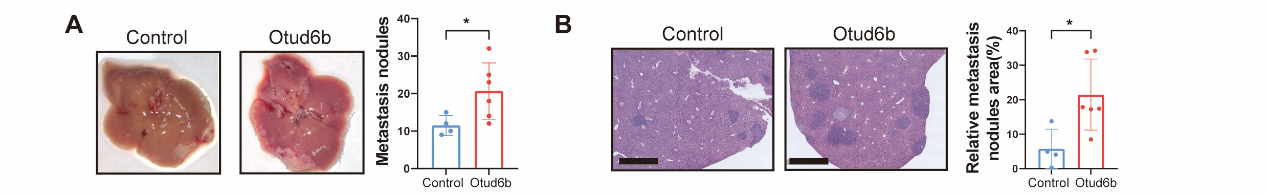


**Figure S2.**

OTUD6B promotes colorectal cancer liver metastasis in nude mice. **A**, Representative images of liver metastatic tissue and the number of liver metastatic nodules from MC38 cells with and without Otud6b overexpression in nude mice (n = 4-6). **B,** Representative pictures and quantitative results of H&E staining from (A). Scale bar, 1 mm. All data values were expressed as the mean ± SEM. A two-side Student’s t test was used for the statistical analysis. *, P<0.05.


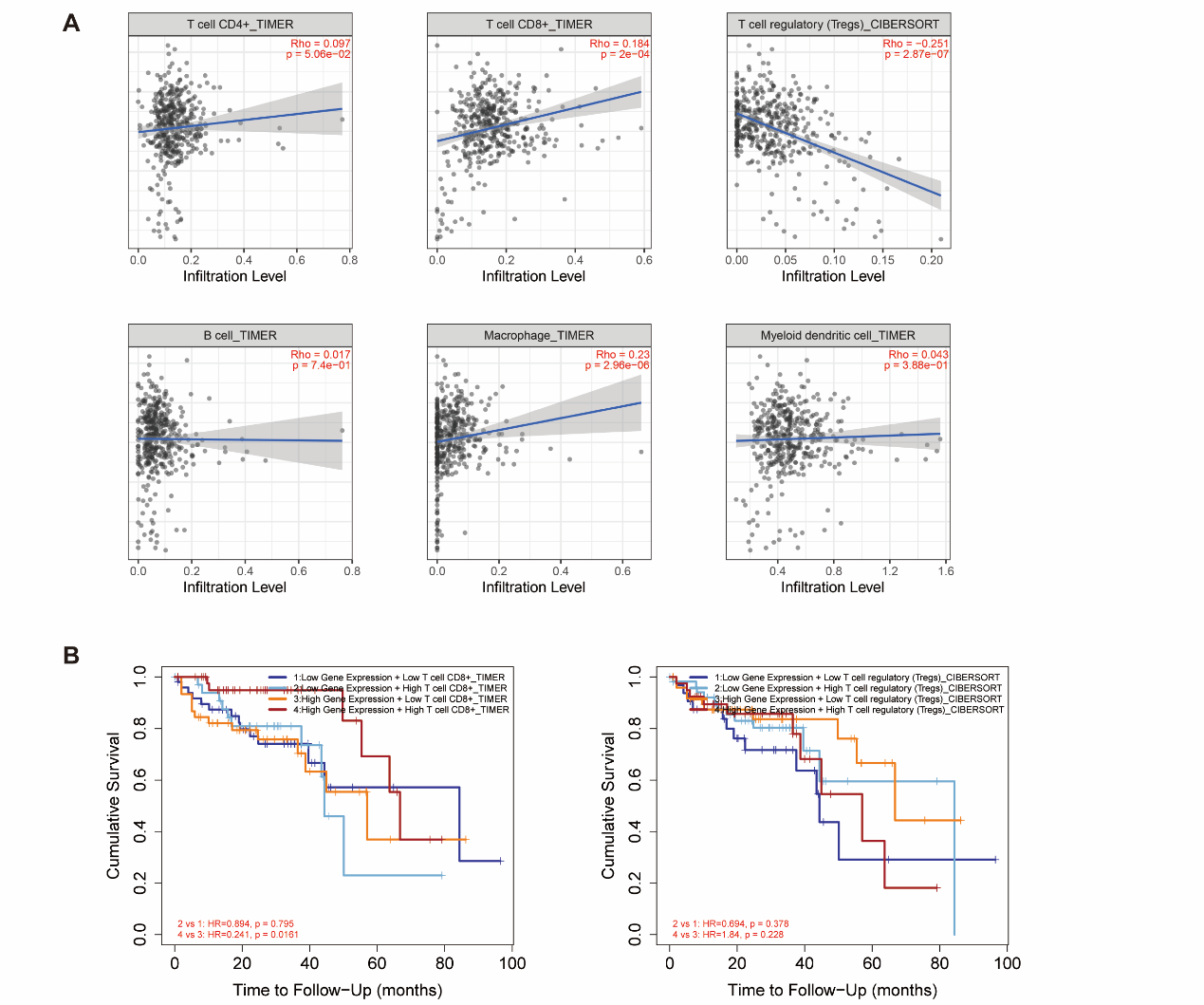


**Figure S3.**

OTUD6B is positively correlated with CD8^+^ T cells. **A,** The relationship between OTUD6B expression and immune cell was analyzed by TIMER database. **B,** Overall survival analysis between OTUD6B expression and T cell in COAD patients by TIMER database.


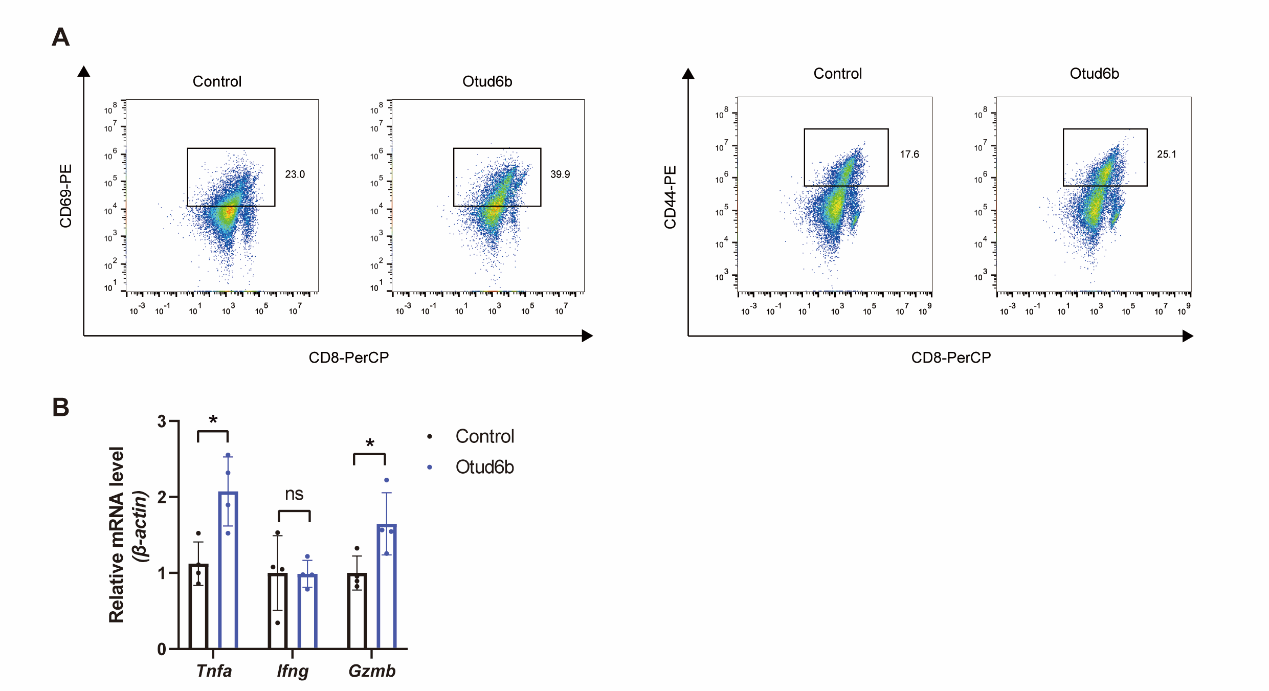


**Figure S4.**

Otud6b promotes CD8^+^ T cell activation in C57BL/6J mice with CRLM. **A,** Representative flow cytometry plots and quantification of CD69^+^ CD8^+^ or CD44^+^ CD8^+^ T cells for the indicated groups. **B,** The relative mRNA levels of *Tnfa*, *Ifng* and *Gzmb* from liver tissues injected with Control or MC38-Otud6b cells in C57BL/6J mice (n=4). All data values were expressed as the mean ± SEM. A two-side Student’s t test was used for the statistical analysis. *, P<0.05; ns, not significant.


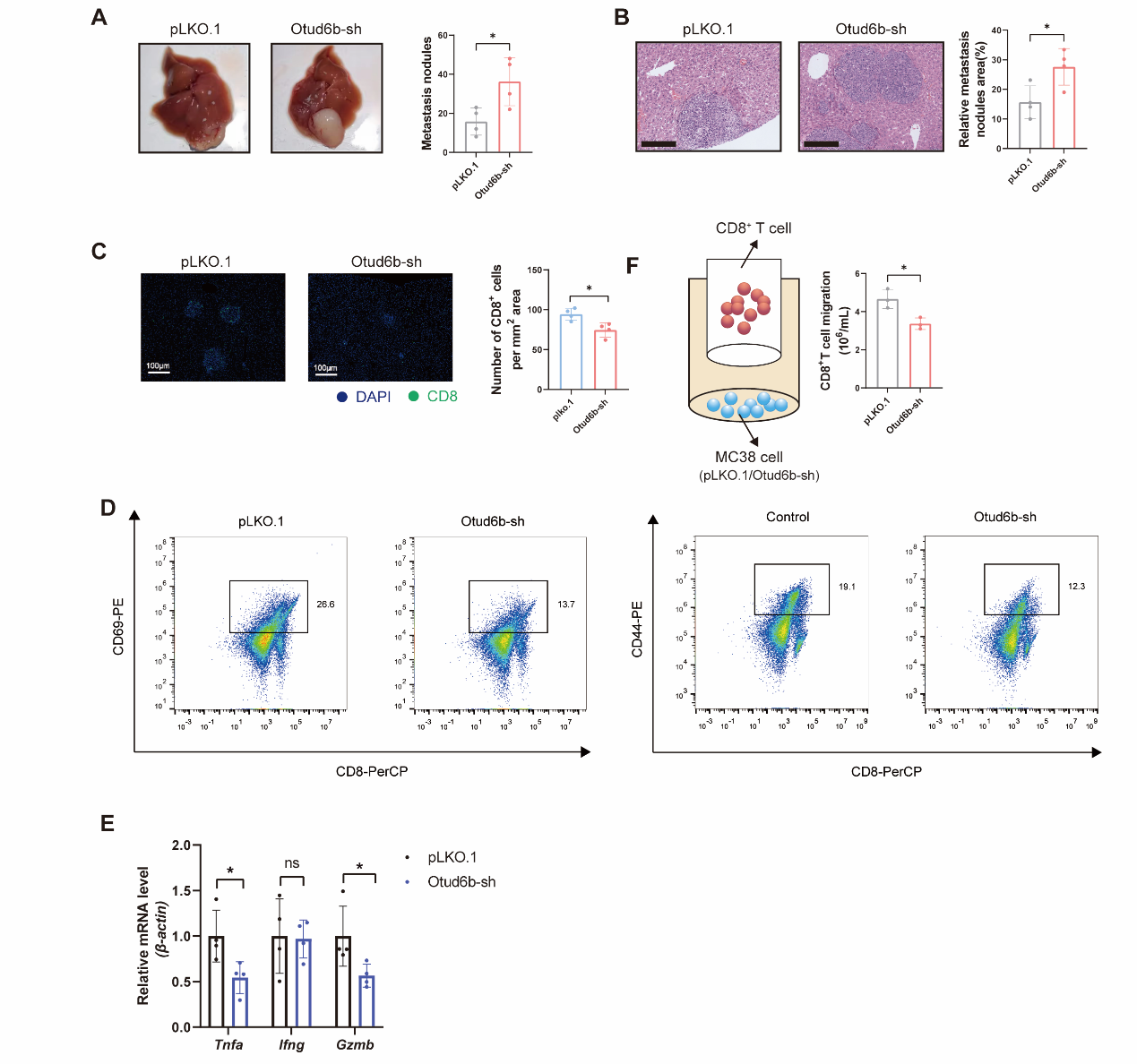


**Figure S5.**

OTUD6B knockdown inhibits CD8^+^ T cell infiltration and promotes CRLM in C57BL/6J mice. **A,** Representative pictures and quantitative results of liver metastatic nodules from C57BL/6J mice injected with MC38-pLKO.1 or MC38-Otud6b-sh cells (n = 4). **B,** Representative pictures and quantitative results of H&E staining from (A). Scale bar, 1 mm. **C,** Representative flow cytometry plots and quantification of CD3^+^ CD8^+^ T cells for the indicated groups. **D,** Representative flow cytometry plots and quantification of CD69^+^ CD8^+^ or CD44^+^ CD8^+^ T cells for the indicated groups. **E,** The relative mRNA levels of *Tnfa*, *Ifng* and *Gzmb* from liver tissues injected with MC38-pLKO.1 or MC38-Otud6b-sh cells in C57BL/6J mice (n=4). **F,** The schematic diagram of CD8^+^ T cell migration test (left). Relative migration of mouse CD8^+^ T cells co-incubated with culture medium supernatant from pLKO.1 or Otud6b-sh MC38 cells (right) (n = 3). All data values were expressed as the mean ± SEM. A two-side Student’s t test was used for the statistical analysis. *, P<0.05; ns, not significant.


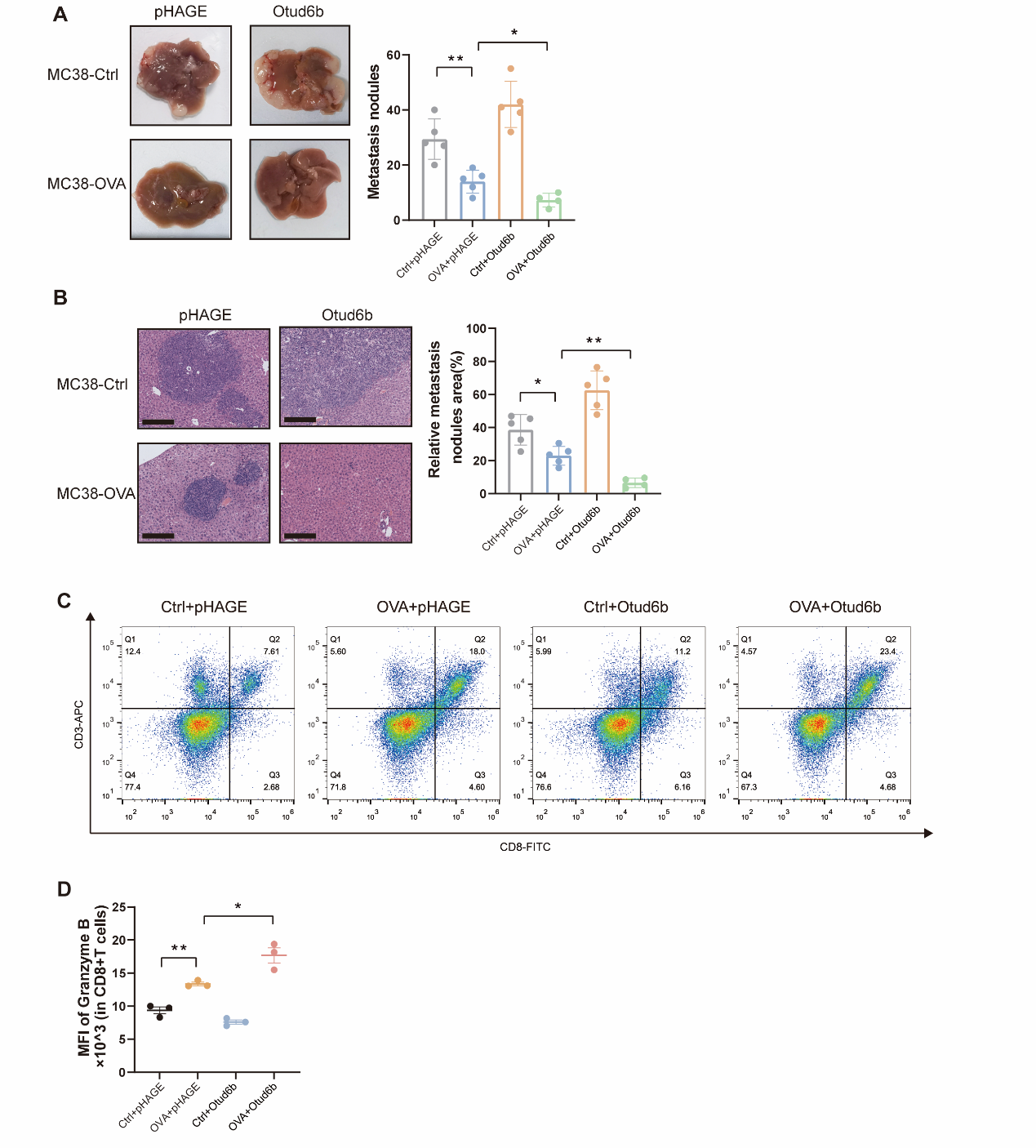


**Figure S6**

OTUD6B inhibits CRLM when CD8^+^ T cells are activated. **A,** Representative pictures and quantitative results of liver metastatic nodules from OT-1 mice with or without OVA (n = 4-5). **B,** Representative pictures and quantitative results of H&E staining from (A). Scale bar, 1 mm. **C,** Representative flow cytometry plots and quantification of CD3^+^ CD8^+^ T cells for the indicated groups. **D,** The statistical data for Granzyme B^+^ CD8^+^ T cells in the liver cancer tissues. All data values were expressed as the mean ± SEM. A two-side Student’s t test was used for the statistical analysis. *, P<0.05; **, P< 0.01.


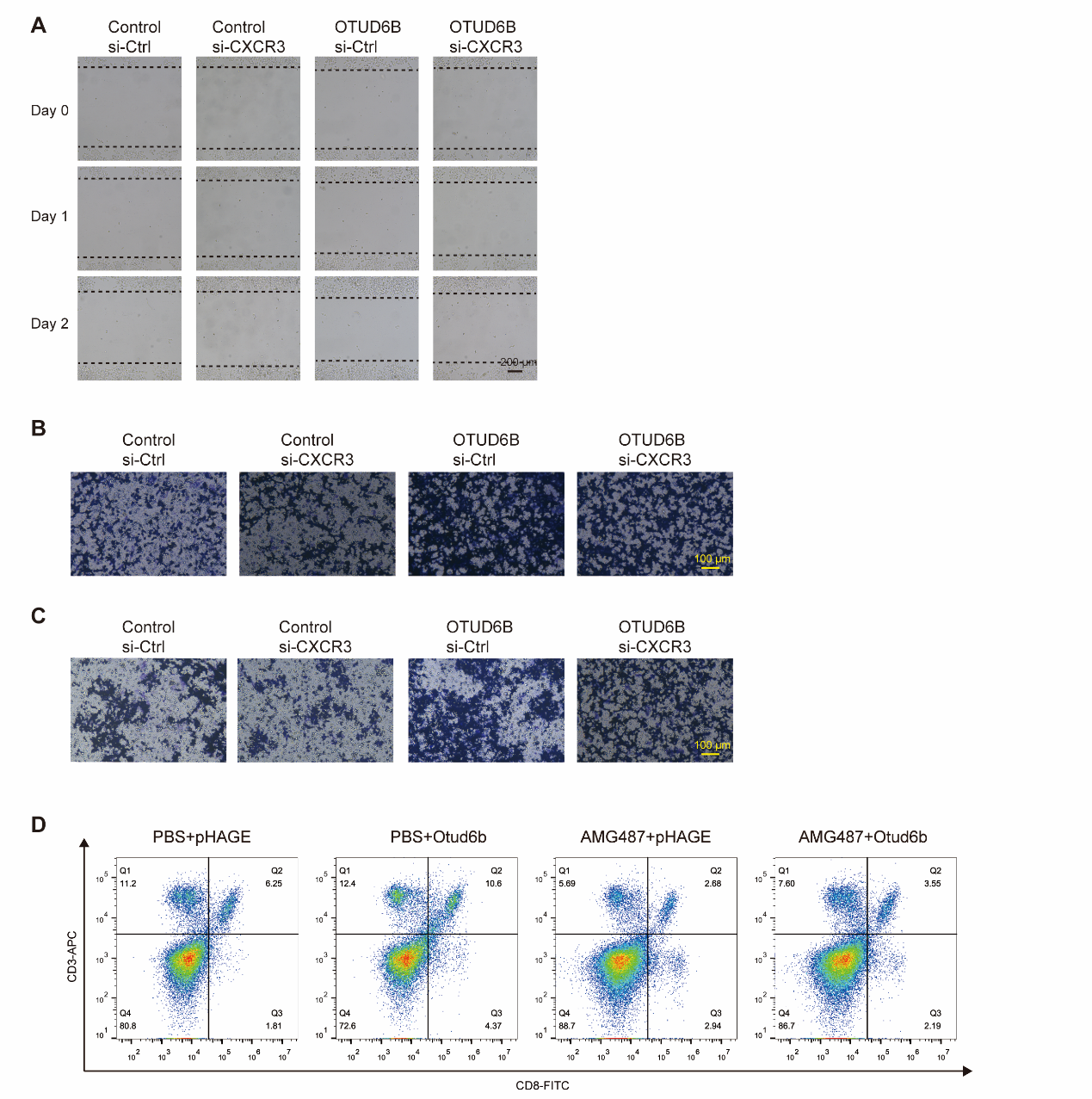


**Figure S7**

OTUD6B regulates CRLM through CXCL11-CXCR3 axis. **A,** Effects of CXCR3 knockdown on the migration of HCT116 cells sing a wound-healing assay. Wound closure was determined at 1- and 2-d time points. Scale bar, 200 μm. **B,** Effects of CXCR3 knockdown on cell migration. Scale bar, 100 μm. **C,** Effects of CXCR3 knockdown on cell invasion. Scale bar, 100 μm. **D,** Representative flow cytometry plots and quantification of CD3^+^ CD8^+^ T cells for the indicated groups.


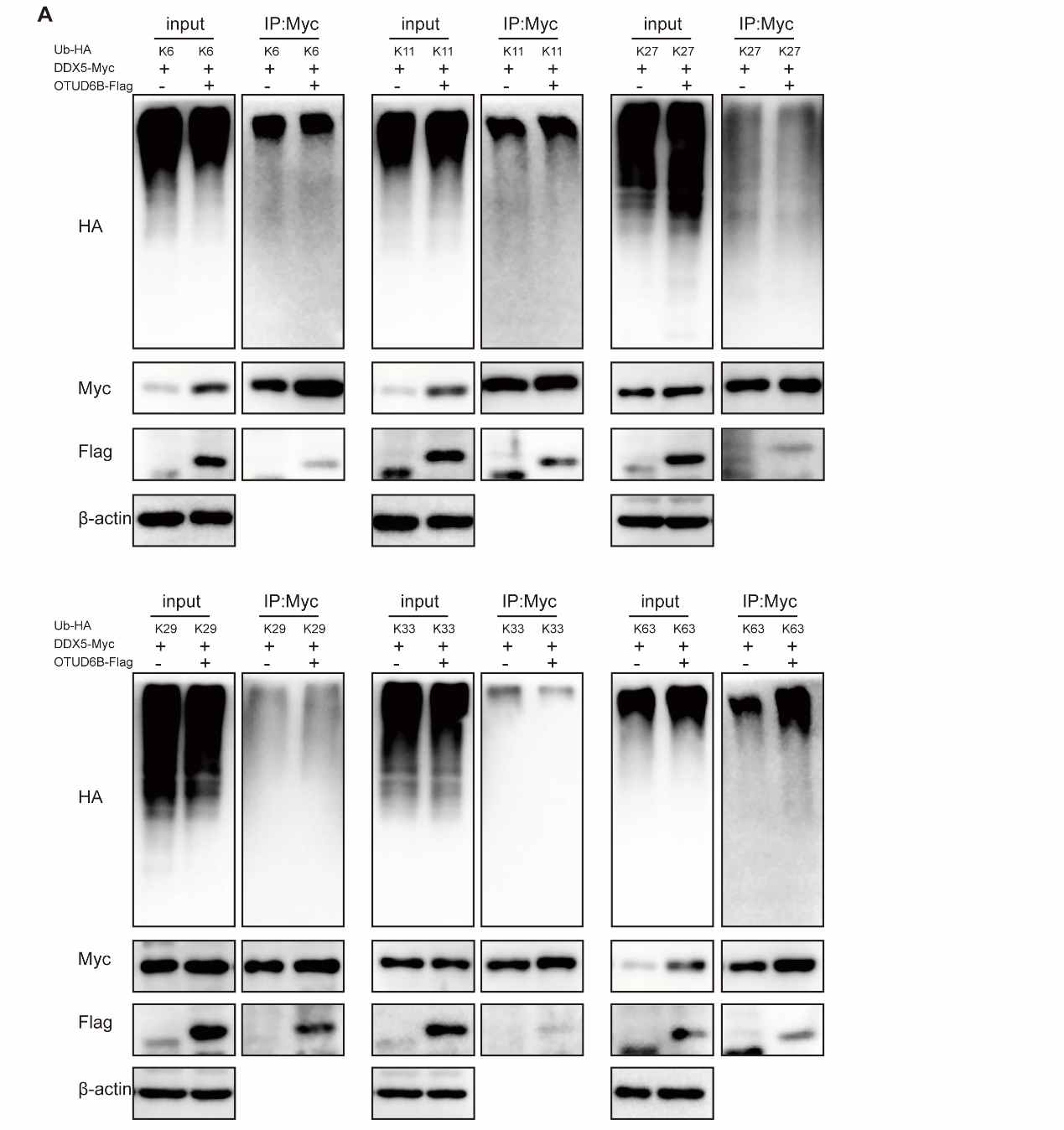


**Figure S8.**

The effect of OTUD6B on different types of ubiquitination. **A,** HCT116 cells were transfected with the indicated plasmids, followed by treatment with MG132 (5 μM) for 12 h prior to collection. The lysates were incubated with Myc antibody and then subjected to immunoblotting.


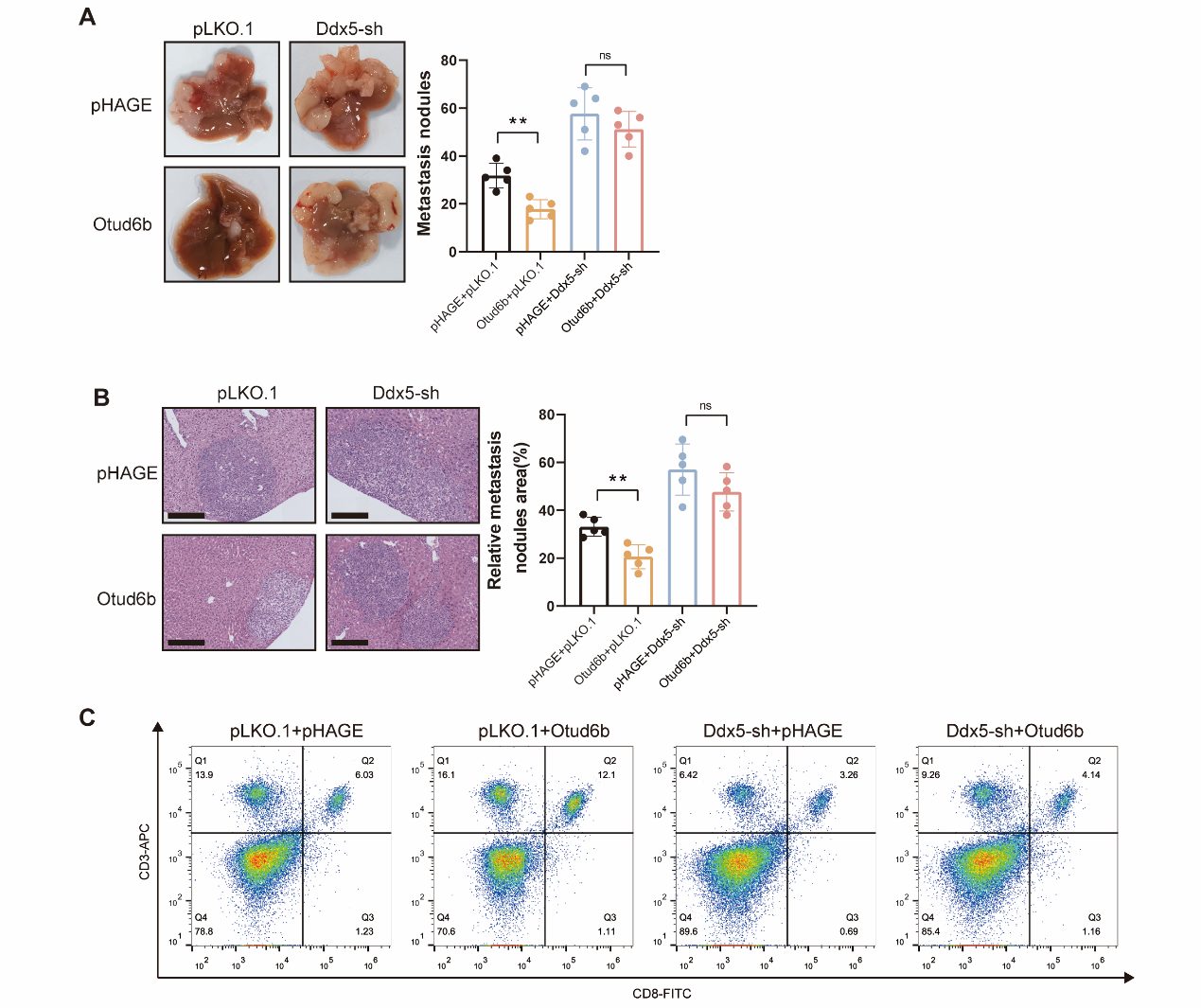


**Figure S9.**

OTUD6B inhibits colorectal cancer liver metastasis through DDX5. **A,** Representative pictures of liver metastatic tissue and the number of liver metastatic nodules from C57BL/6J mice injected with indicated MC38 cells (n=5). **B,** Representative pictures and quantitative results of H&E staining from (A). Scale bar, 1 mm. **C,** Representative flow cytometry plots and quantification of CD3^+^ CD8^+^ T cells for the indicated groups. All data values were expressed as the mean ± SEM. A two-side Student’s t test was used for the statistical analysis. **, P< 0.01; ns, no significant.


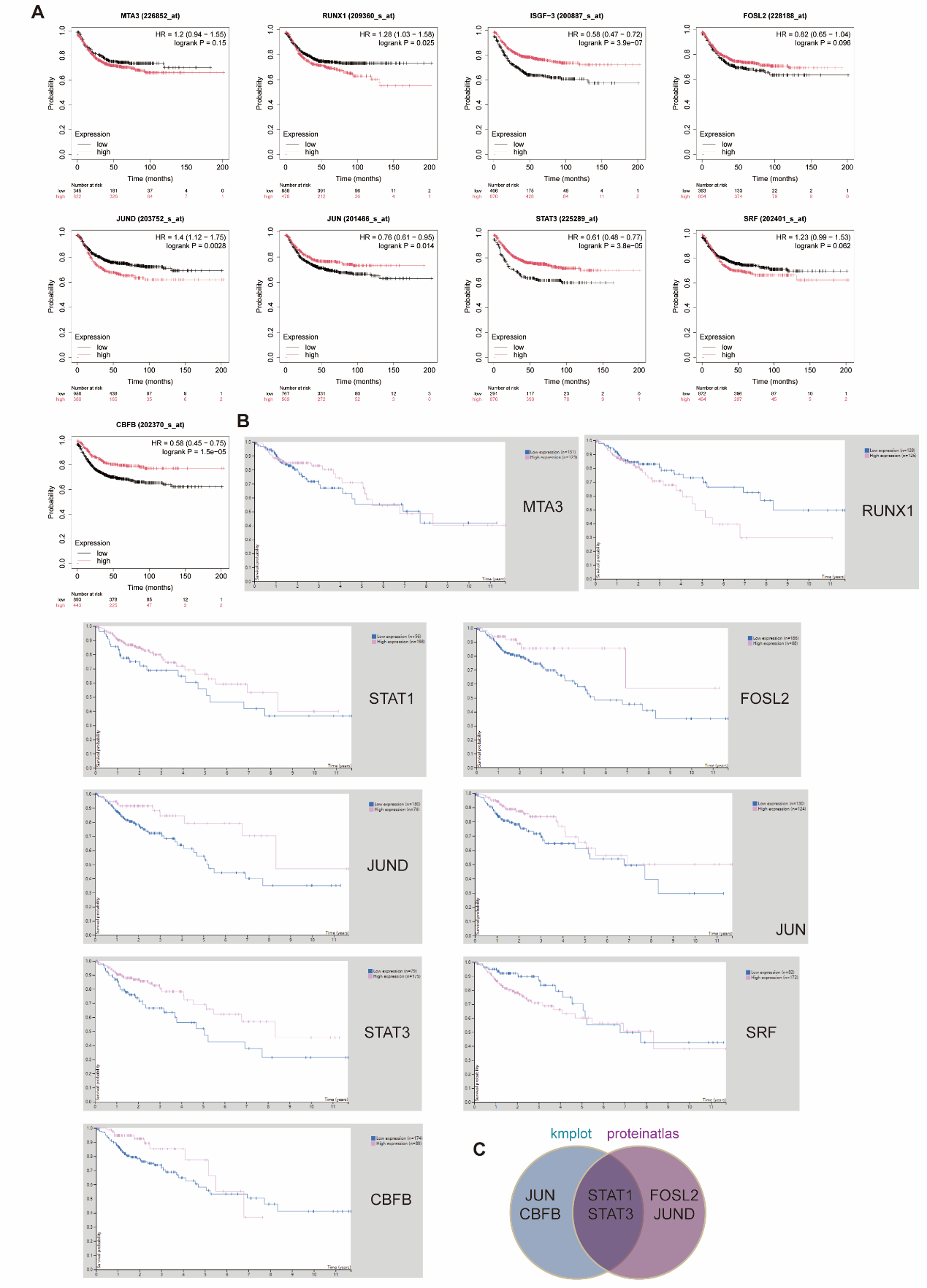


**Figure S10.**

Survival curve of transcription factors in CRC. **A,** Survival curve of transcription factors in CRC using kmplot database. **B,** Survival curve of transcription factors in CRC using proteinatlas database. **C,** In the two databases, there were 4 positive correlations between gene expression and survival, and 2 in the intersection of the two databases.

**Table S1**

Antibody used in this study

| **Name** | **Brand** | **Code** | **RRID** |
| --- | --- | --- | --- |
| OTUD6B | Novus Biologicals | NBP1-85652 | AB_11010410 |
| OTUD6B | Abclonal | A14511 | AB_2761386 |
| DDX5 | Abclonal | A11339 | AB_2861546 |
| STAT3 | Abclonal | A1192 | AB_2861642 |
| β-actin | Cell Signaling Technology | 4967S | AB_330288 |
| GAPDH | Cell Signaling Technology | 8146S | AB_10950495 |
| Myc | Cell Signaling Technology | 2276S | AB_331783 |
| Flag | Cell Signaling Technology | 8146S | AB_10950495 |
| HA | Cell Signaling Technology | 2367S | AB_10691311 |
| CD3 | Elabscience | E-AB-F1013UE | AB_3065041 |
| CD8 | Elabscience | E-AB-F1104UC | NA |
| Granzyme B | BioLegend | 372207 | NA |

**Table S2**

Primer sequences for qPCR used in this study.

| **Gene** | **Forward sequence** | **Reverse sequence** |
| --- | --- | --- |
| OTUD6B | CTGCTGAGAAGGCATCGCAAAG | GCCACATCTTCGGTGAGTTGCT |
| DDX5 | GCTTCCTGCCATTGTCCACATC | GCAGCTACTTGCTGCACCTGTT |
| STAT3 | CTTTGAGACCGAGGTGTATCACC | GGTCAGCATGTTGTACCACAGG |
| STAT1 | ATGGCAGTCTGGCGGCTGAATT | CCAAACCAGGCTGGCACAATTG |
| CXCL11 | GGTGAGAAGAGATGTCTGAATCC | GTCCATCCTTGGAAGCACTGCA |
| CXCR3 | ACGAGAGTGACTCGTGCTGTAC | GCAGAAAGAGGAGGCTGTAGAG |
| CCL2 | TCCTGAACCCACTTCTGCTTGG | AAGTTGTCTGTGTGCGCAAATCC |
| CCL5 | CCTGCTGCTTTGCCTACATTGC | ACACACTTGGCGGTTCTTTCGG |
| CCL20 | AAGTTGTCTGTGTGCGCAAATCC | CCATTCCAGAAAAGCCACAGTTTT |
| CCL22 | TCCTGGGTTCAAGCGATTCTCC | GTCAGGAGTTCAAGACCAGCCT |
| CXCL8 | GAGAGTGATTGAGAGTGGACCAC | CACAACCCTCTGCACCCAGTTT |
| CXCL9 | CTGTTCCTGCATCAGCACCAAC | TGAACTCCATTCTTCAGTGTAGCA |
| CXCL10 | GGTGAGAAGAGATGTCTGAATCC | GTCCATCCTTGGAAGCACTGCA |
| IL-17 | CGGACTGTGATGGTCAACCTGA | GCACTTTGCCTCCCAGATCACA |
| β-actin | CACCATTGGCAATGAGCGGTTC | AGGTCTTTGCGGATGTCCACGT |
| GAPDH | GTCTCCTCTGACTTCAACAGCG | ACCACCCTGTTGCTGTAGCCAA |
