# Supplementary material for: ATRA upregulates OTUD6B to recruit CD8+ T cells to suppress colorectal liver metastasis by stabilizing DDX5/STAT3/CXCL11 axis
Source: Cell Death Dis. 2025 Jul 12;16(1):521. doi: 10.1038/s41419-025-07837-0 (PMC12255723; doi:10.1038/s41419-025-07837-0)

Fig 1E

OTUD6B

β-actin

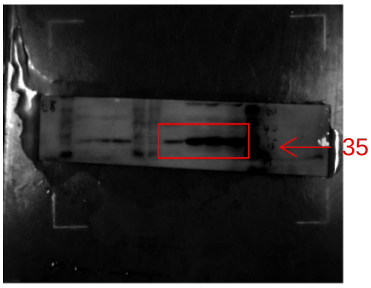

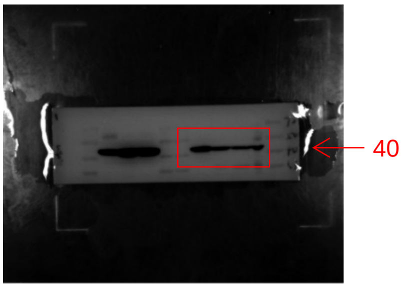

Fig 1F

OTUD6B

β-actin

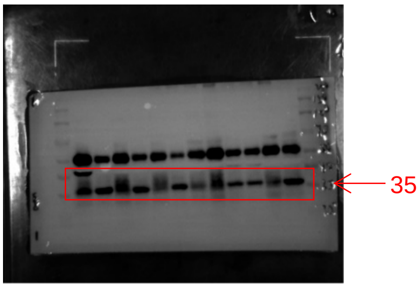

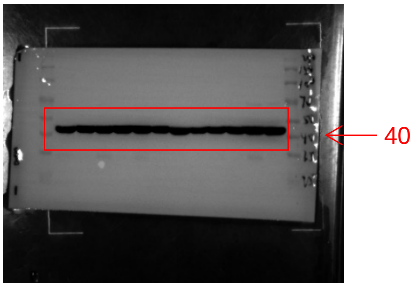

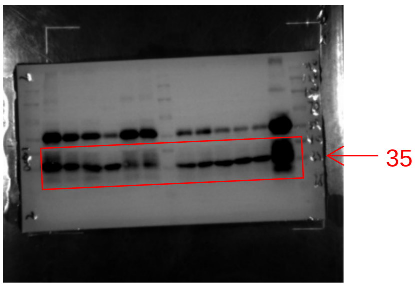

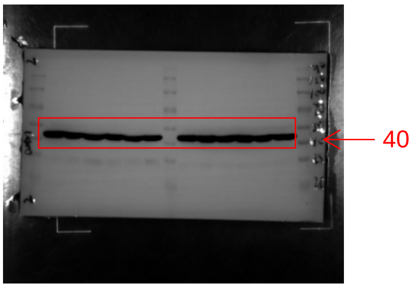

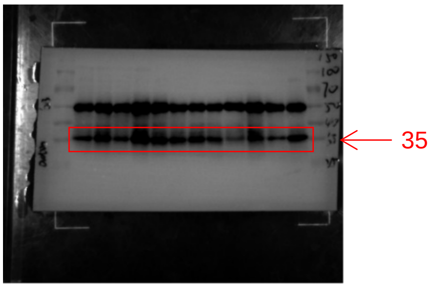

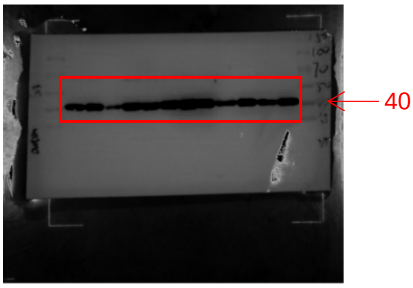

Fig 2A

Flag-OTUD6B

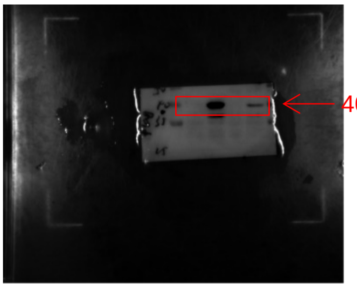

GAPDH

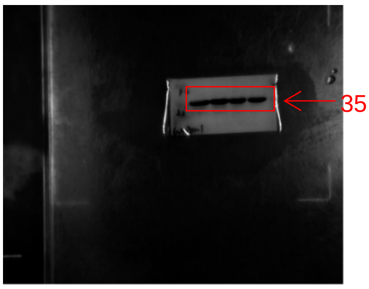

Fig 5D

myc-DDX5

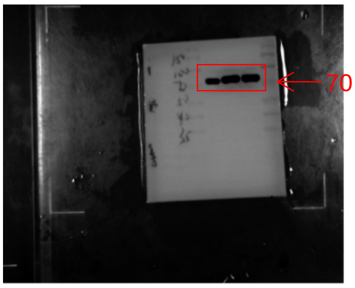

Flag-OTUD6B

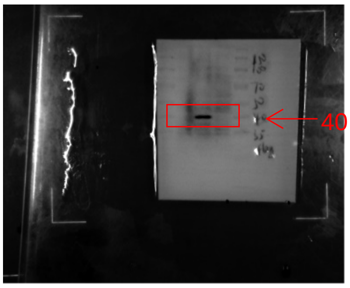

β-actin

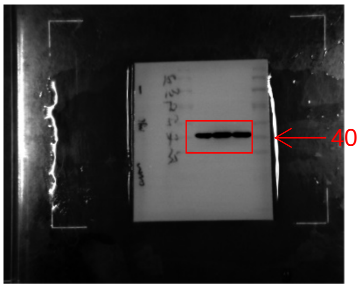

Fig 5E

myc-DDX5

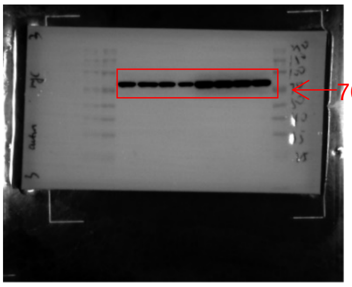

Flag-OTUD6B

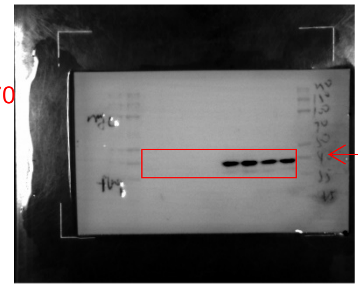

β-actin

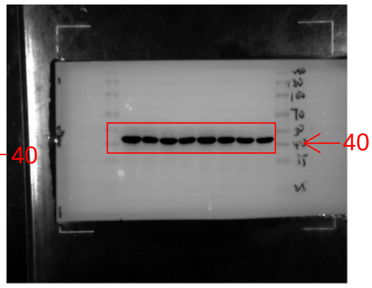

Fig 5F

myc-DDX5 | Flag-OTUD6B | β-actin

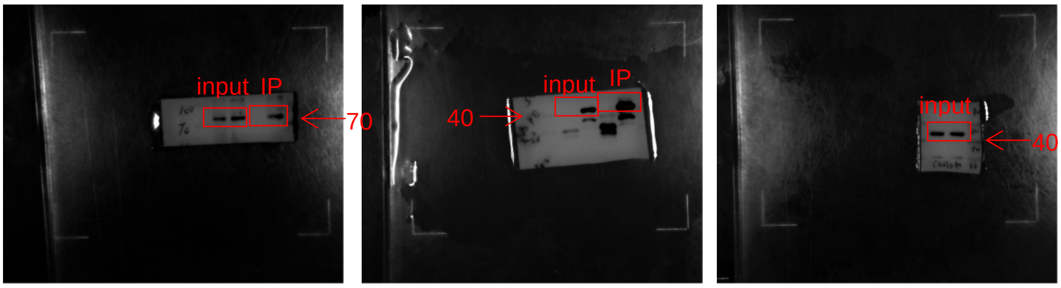

Fig 5G

myc-DDX5 | Flag-OTUD6B | β-actin

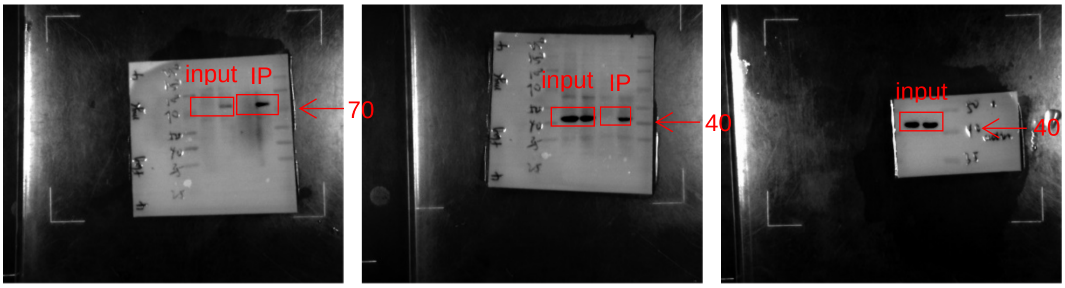

Fig 5H

myc-DDX5 | Flag-OTUD6B | β-actin

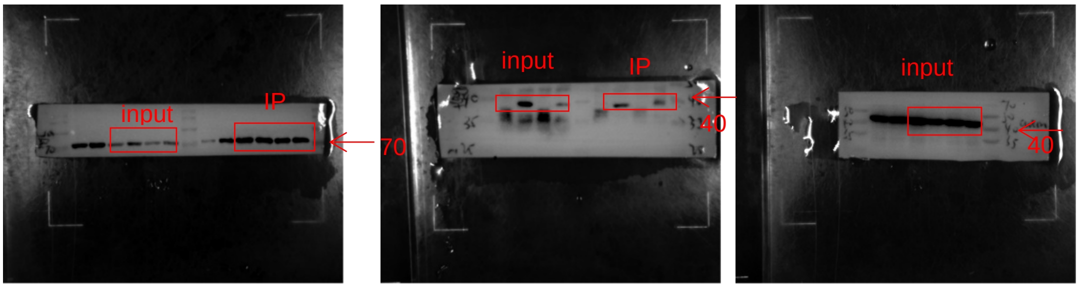

HA (input)

HA （IP）

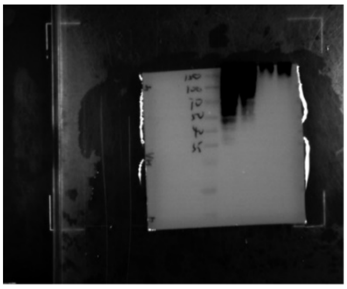

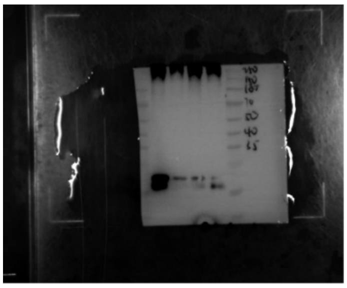

Fig 6E

STAT3

DDX5

β-actin

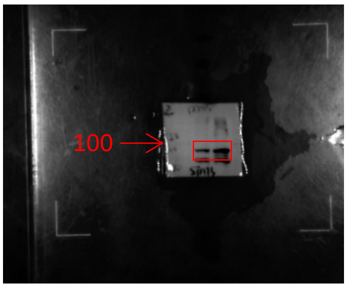

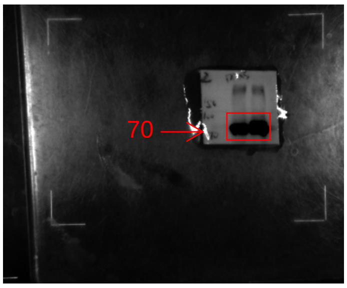

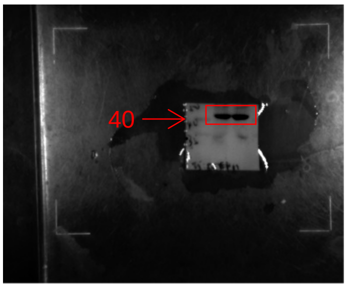

Fig 7B

OTUD6B

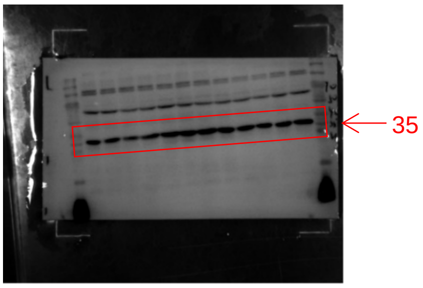

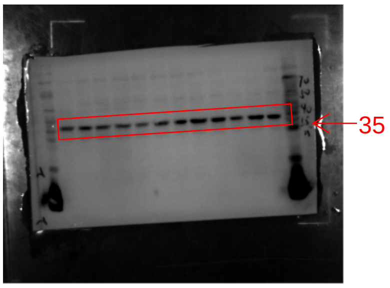

DDX5

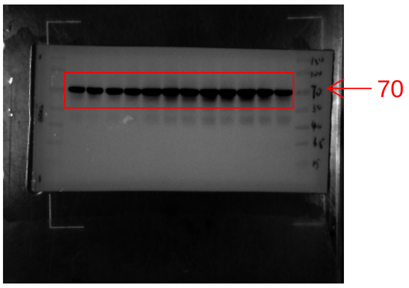

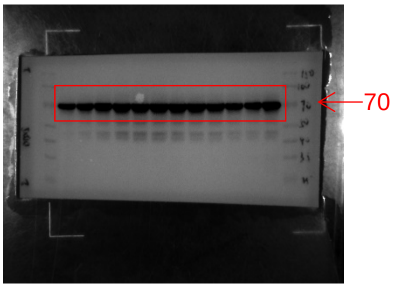

β-actin

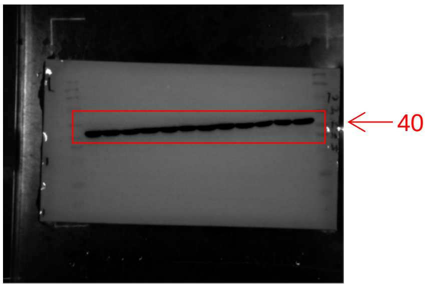

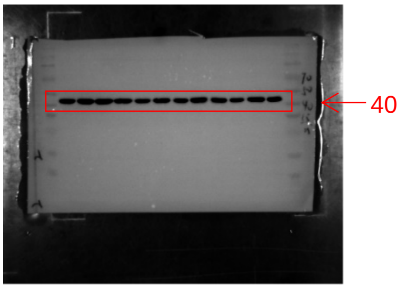

Fig S1A

OTUD6B

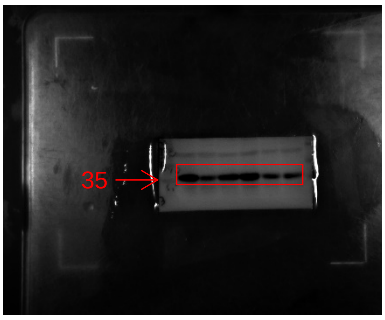

β-actin

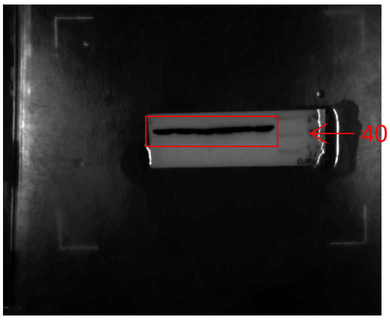

Fig S7A

HA

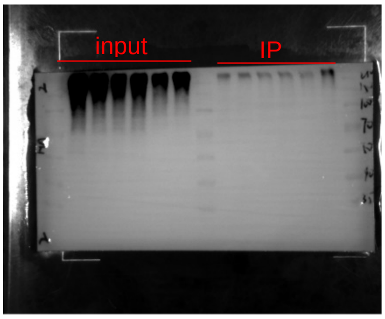

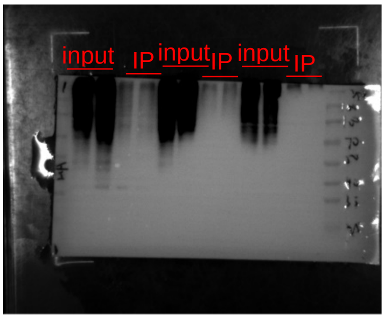

Myc-DDX5

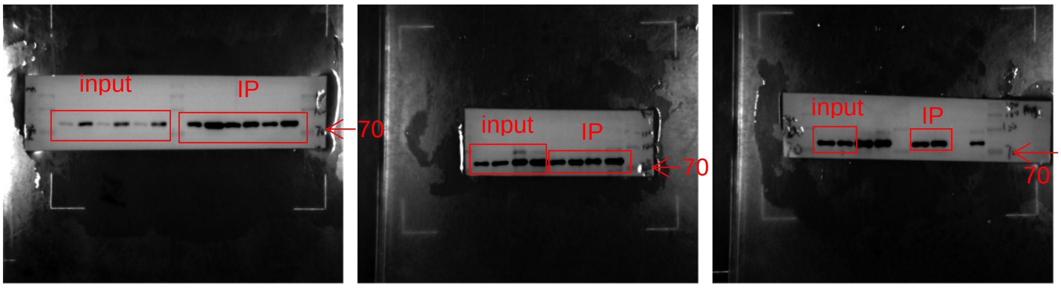

Flag-OTUD6B

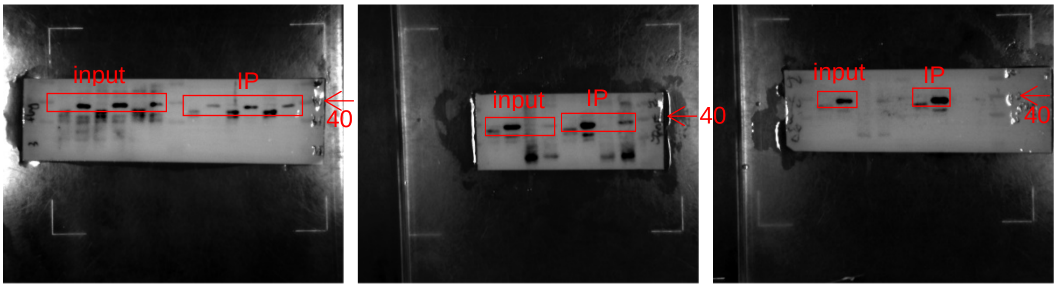

β-actin

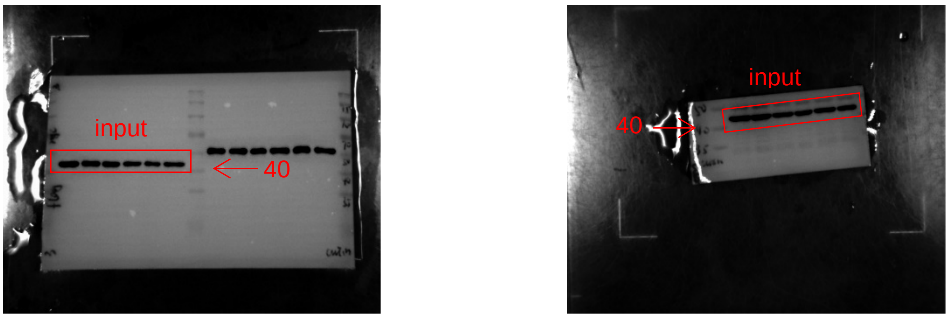

Supplement: Supplementary file 2 — Original Data [file 41419_2025_7837_MOESM2_ESM.pdf]
